# Supplementary material for: Synthetic intrinsically disordered protein fusion tags that enhance protein solubility
Source: Nat Commun. 2024 May 2;15:3727. doi: 10.1038/s41467-024-47519-7 (PMC11066018; doi:10.1038/s41467-024-47519-7)
Supplement: Supplementary file 1 — Supplementary Information [file 41467_2024_47519_MOESM1_ESM.pdf]

Supplementary information

Synthetic intrinsically disordered protein fusion tags that enhance protein solubility

Nicholas C. Tang[#1], Jonathan Su[#1], Yulia Shmidov[#1], Garrett Kelly[1], Sonal Deshpande[1], Parul Sirohi[1], Nikhil Peterson[1], and Ashutosh Chilkoti[1]

[#] Authors contributed equally

[1]Department of Biomedical Engineering, Duke University, Durham, North Carolina, 27708, USA

Corresponding author: Ashutosh Chilkoti- chilkoti@duke.edu

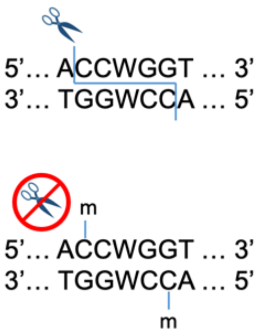

Supplementary Figure 1: SexAI restriction site. The restriction enzyme site encodes a proline-glycine sequence, and enzyme activity is abolished upon methylation within the recognition site**.**

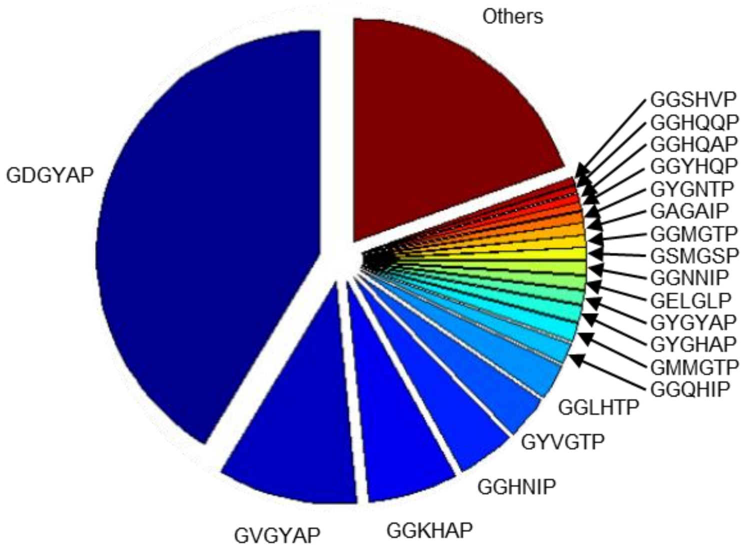

Supplementary Figure 2: Illumina Miseq analysis of cloned library of plasmid DNA verifies the presence of 865 unique motifs.

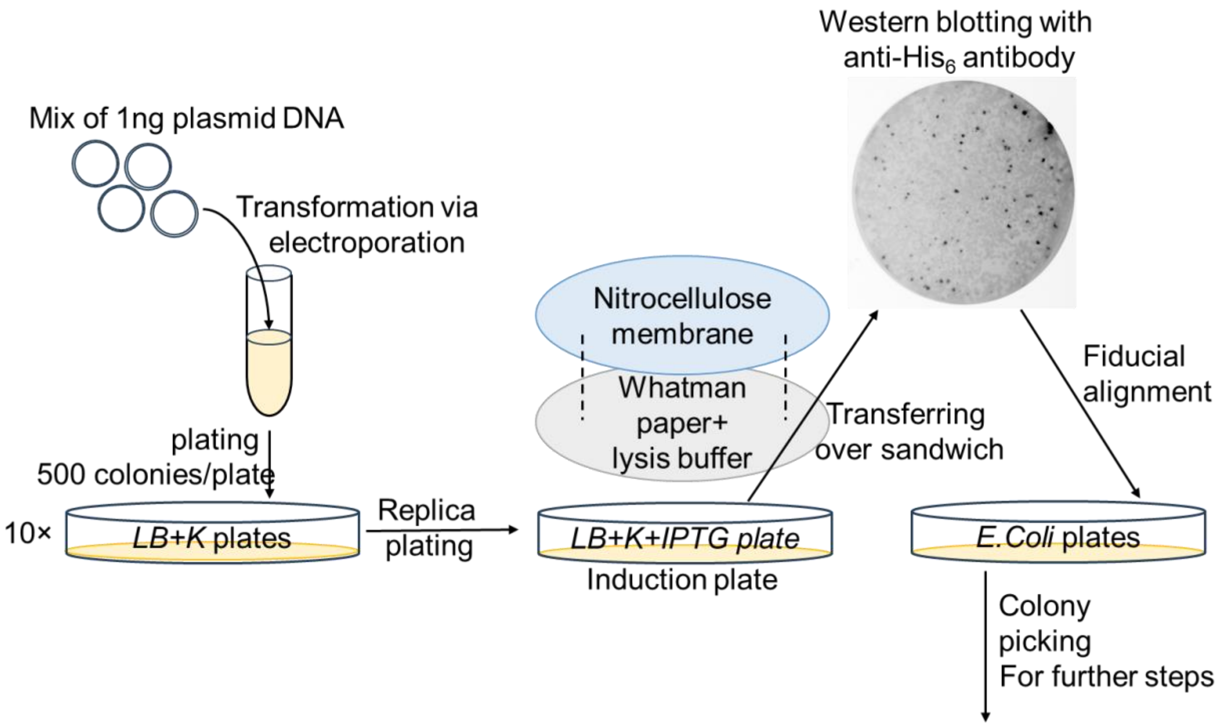

Supplementary Figure 3: Illustration of the steps of the CoFi method. For a full description please see the methods section.

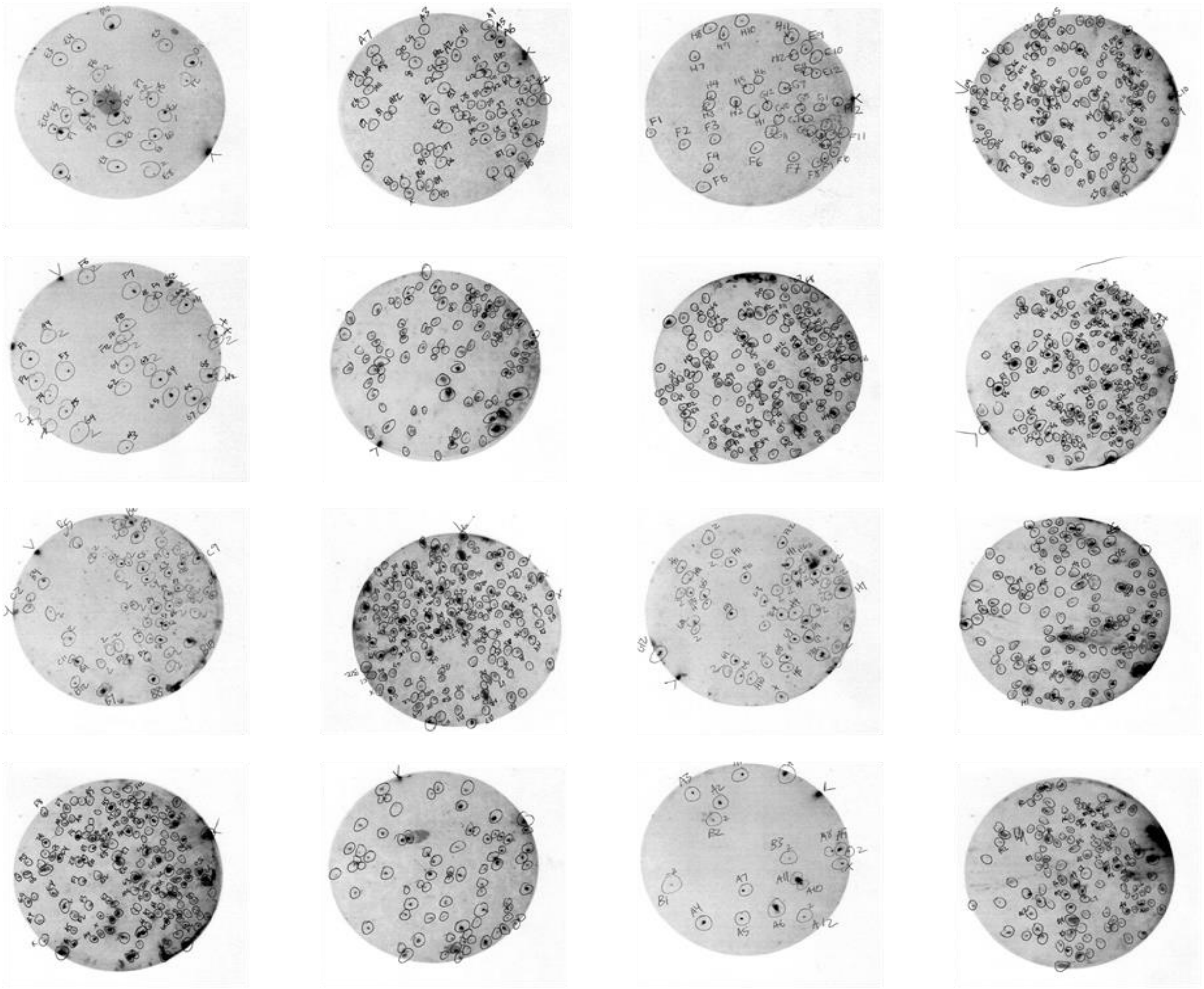

Supplementary Figure 4: Complete set of agar plates where the CoFi method was used to identify and locate *E. coli* colonies expressing soluble proteins. Dark spots on a His$_6$-tag Western blot indicate colonies expressing soluble proteins, and colonies were identified manually from a printed image. Experiment was performed once.

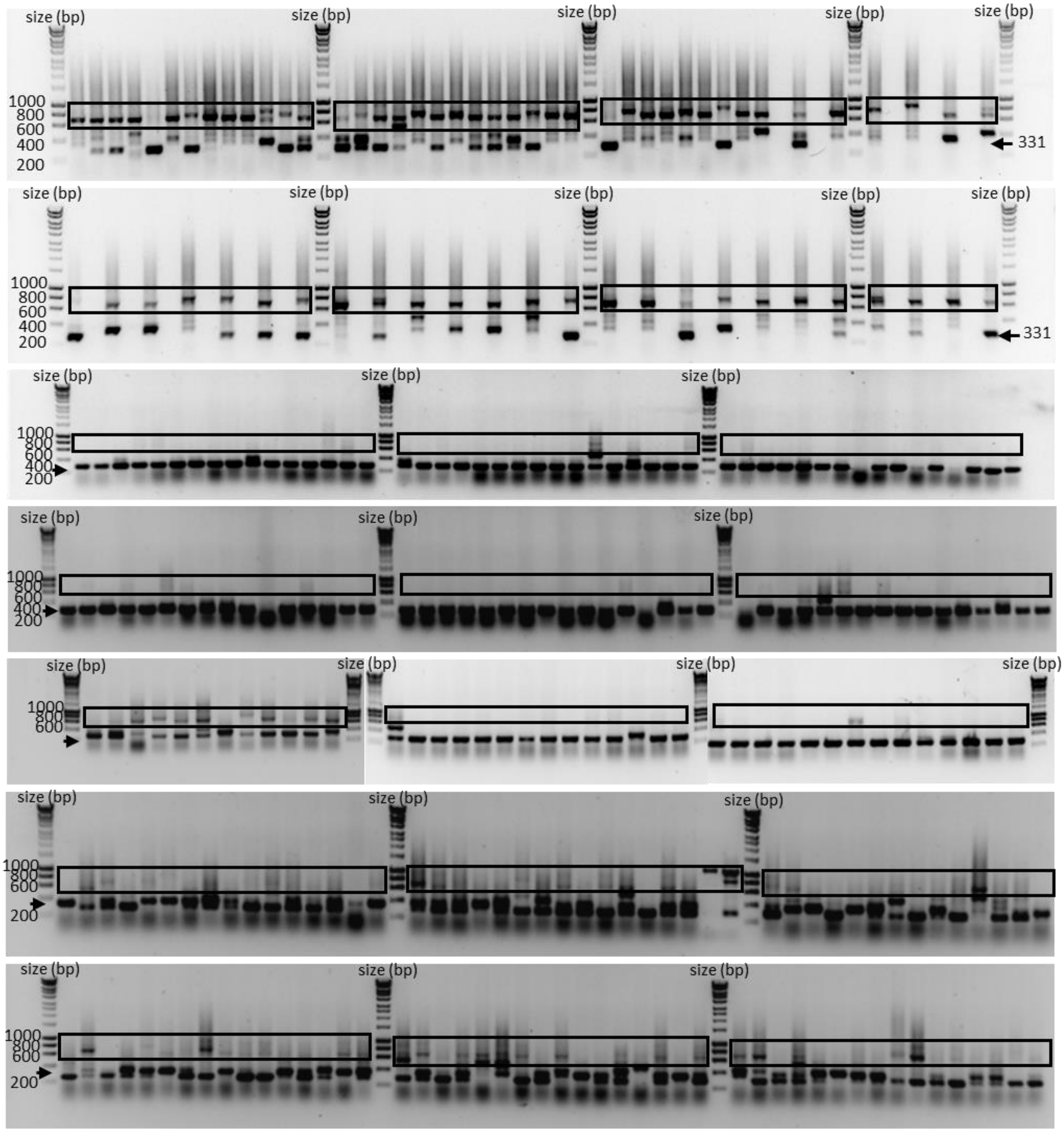

Supplementary Figure 5: Total set of gels showing PCR directly from library-transformed *E. coli* colonies on dense plates visualized on agarose gel electrophoresis. Some colonies (positive) contained amplicons of expected and desired lengths, encoding 20-32 repeats of amino acid motifs (500-1000 bp including flanking regions, marked by black rectangle on the gel). A weaker ladder pattern is typical for PCR of repetitive sequences. Each colony harbors an expression plasmid containing a repetitive gene. Other colonies (negative) contained amplicon of lengths expected to contain coding regions consisting of a single-oligonucleotide plus flanking regions on the plasmid backbone encompassed by the primer sites (331 bp, marked with arrow). Experiment was performed once.

Supplementary Table 1: Summary of Sanger sequencing results.

Motif	*Occurrences in NGS*	*Occurrences in sanger sequencing*	*Mutations*
GLSGSP	6	3	1
GIRGTP	1	1	4
GGMGTP	8	1	1
GTGMEP	9	1	0
GGMGSP	11	1	2
GGTAAP	13	1	1
GNMGSP	20	1	0
GGSQIP	23	1	1
GTLGQP	38	1	0
GHMGTP	171	3	1
GGLSIP	59	1	0
GNIGTP	65	1	0
GGNYAP	66	1	2
GHHGTP	87	1	0
GGQHTP	194	2	0
`	100	1	0
GEGNIP	223	2	1
GGHMIP	106	1	0
GKFGTP	114	1	0
GNGNVP	252	2	1
GGHQQP	605	4	0
GHIGVP	301	2	0
GTHGTP	145	1	0
GAGAIP	654	4	0
GIGQAP	188	1	0
GGMGIP	209	1	2
GTFGTP	219	1	0
GQSGLP	449	2	0
GAVGTP	242	1	0
GGHHAP	272	1	1
GSMGSP	609	2	0
GVIGIP	316	1	0
GGHQAP	345	1	0
GDNGSP	388	1	0
GGNNIP	414	1	1
GGSHVP	1189	3	0
GGLQQP	454	1	0
GGQHIP	1964	5	0
GHEGSP	487	1	0
GGMLAP	5076	13	0

GGHNIP	3826	5	0
GGLHTP	4024	5	0
GYGHAP	2763	3	1
GDGYAP	8094	11	1
GYGYAP	1450	1	0
GYMGKP	1558	1	0
GGFMQP	2975	2	0
GGKHAP	9432	7	2
GYVGTP	5938	3	1
GELGLP	4028	1	0
GGKHAP	12470	6	0
GVGYAP	5099	1	0

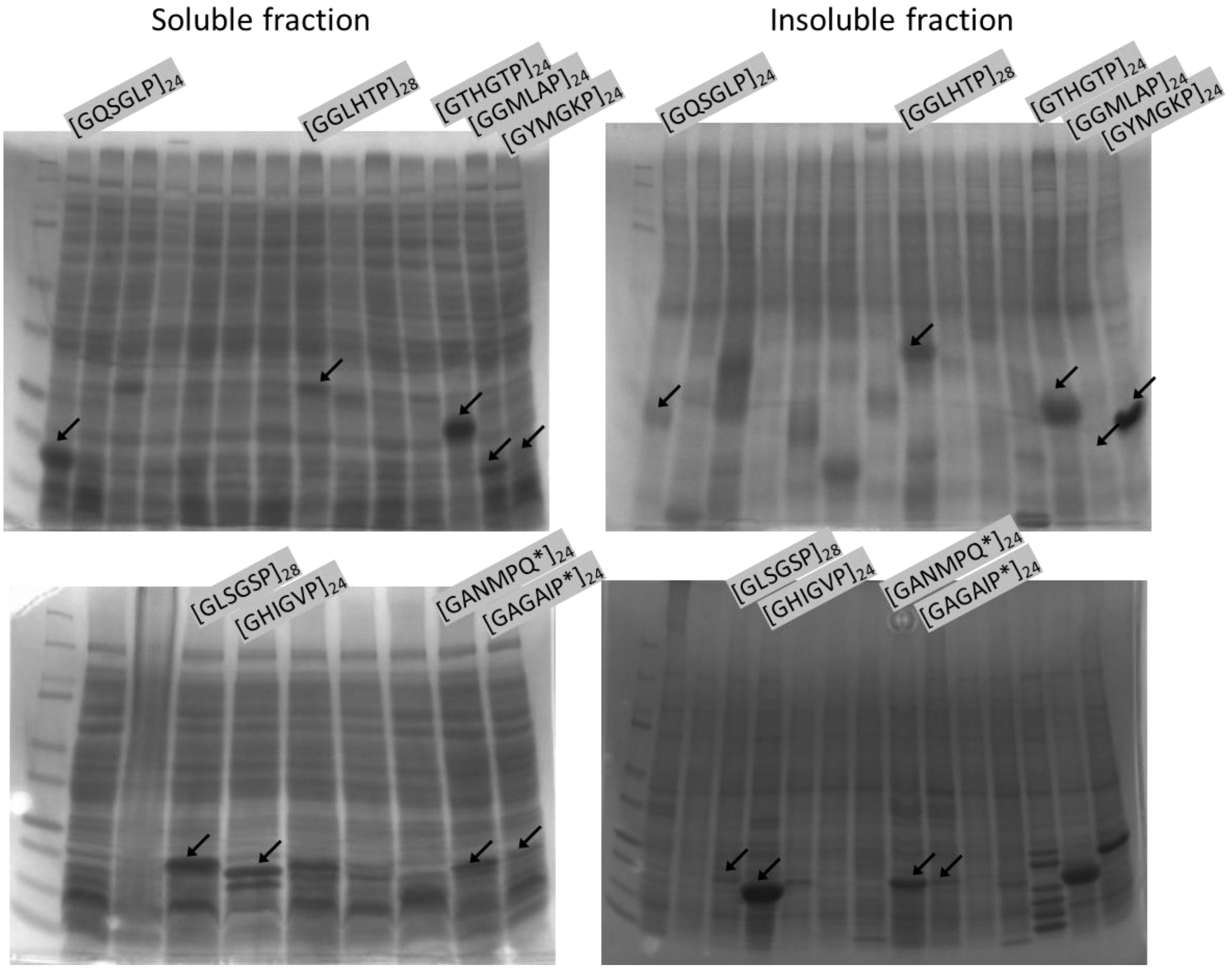

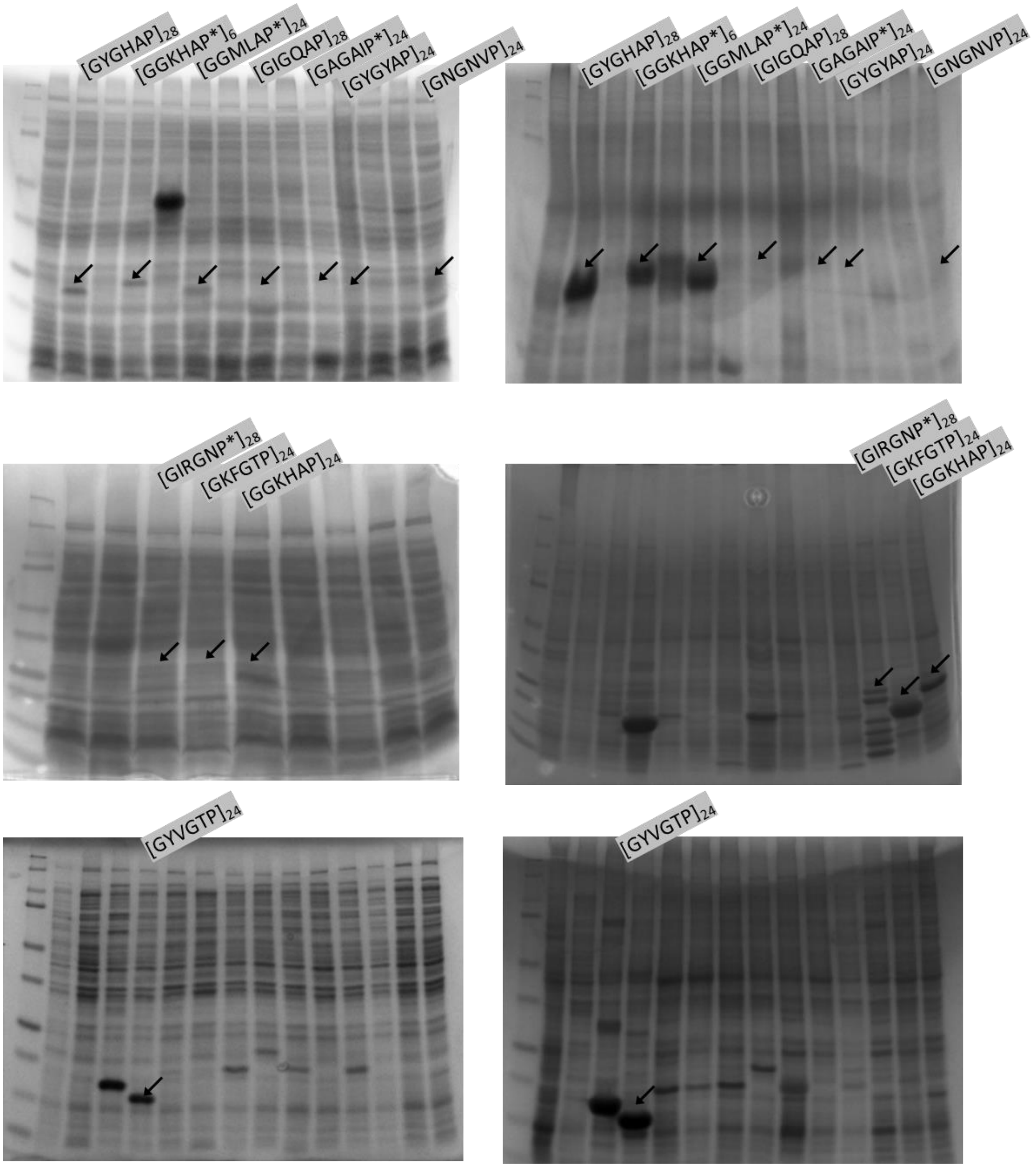

Supplementary Figure 6: Analysis of soluble (left) and insoluble (right) lysate fractions to determine soluble expression of the SynIDPs by SDS-PAGE. The bands marked by arrows indicate the locations of target proteins. Solubility was determined by the intensity of the soluble fraction relative to the insoluble fraction in both dot blot and PAGE gels. Prestained Kaleidoscope™ ladder was used as a molecular weight standard for all gels. The sequences of unlabeled lanes were ignored and discarded for a variety of reasons, but usually due to the presence of duplicates or the harboring of mixtures of genes due to cross-contamination of neighboring

colonies during the colony picking step. Asterix's (*) indicate missense mutations that slightly modify the amino acid sequence. Refer to table summary of the solubility of IDPs and TdT fusion proteins (Table 1) for full amino acid sequences of the SynIDPs. Experiment was performed once.

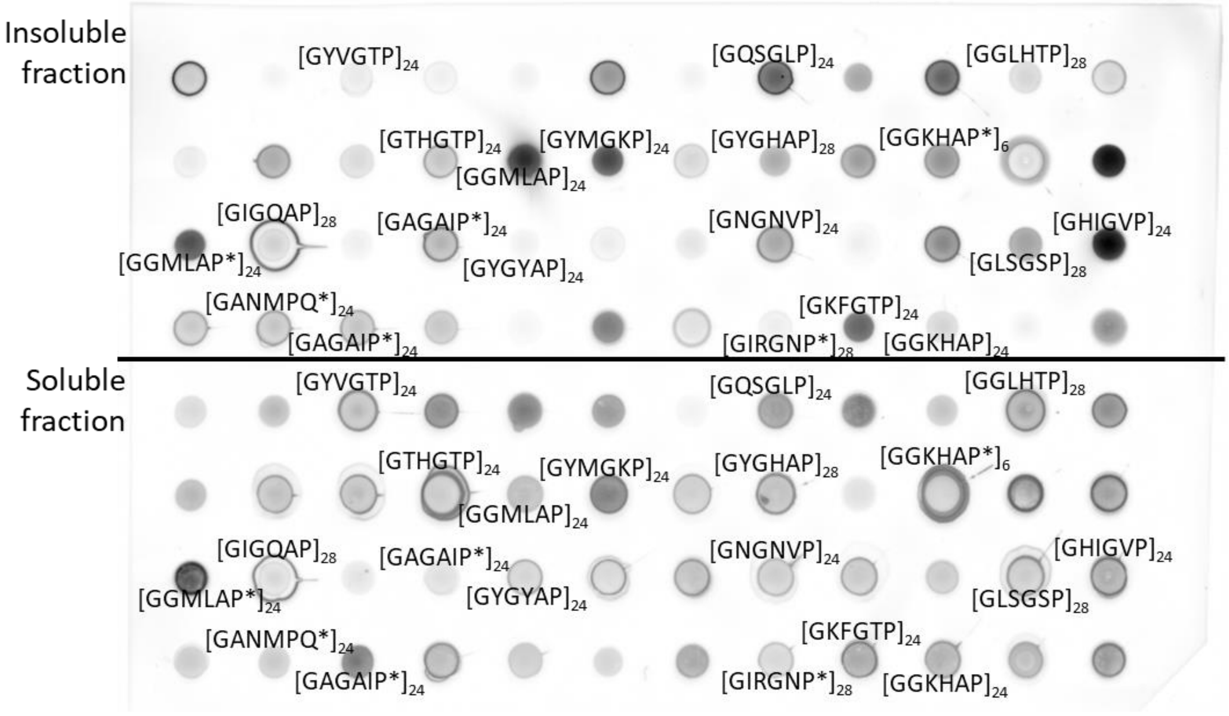

Supplementary Figure 7: Labeled dot blot of soluble (bottom) and insoluble (top) lysate fractions to determine SynIDP solubility. Soluble expression of the SynIDPs was assessed by the intensity of the soluble fraction relative to the insoluble fraction in both dot blot and PAGE gels. The sequences of unlabeled dots were ignored and discarded due to the presence of duplicates or the harboring of mixtures of genes due to cross-contamination of neighboring colonies during the colony picking step. Experiment was performed once.

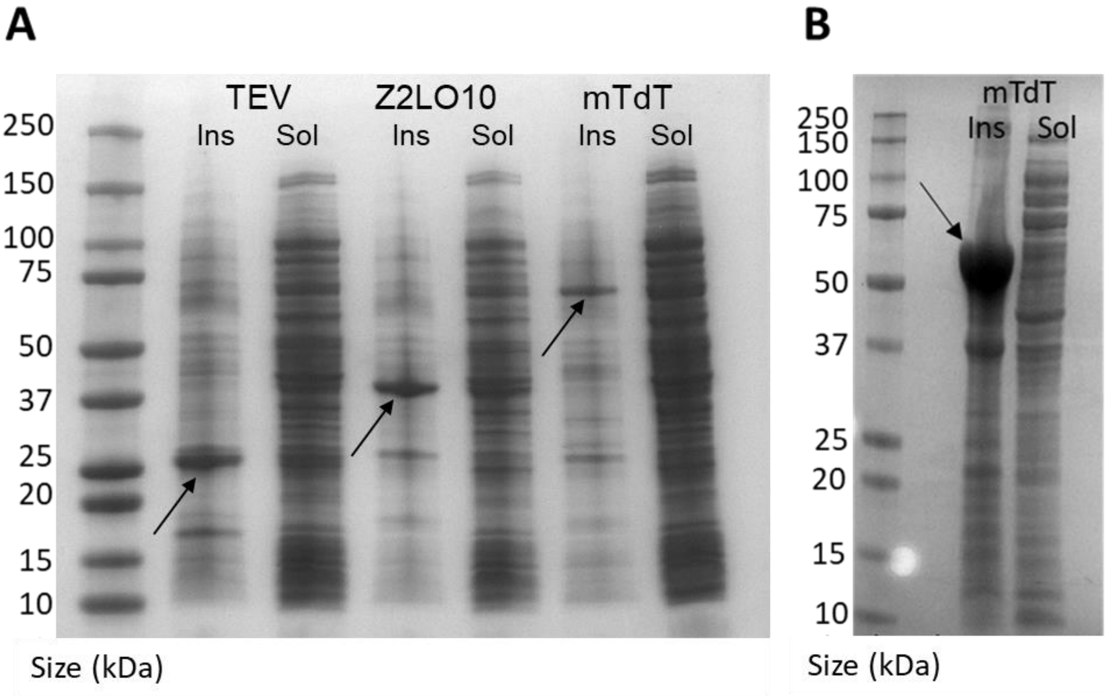

Supplementary Figure 8: Expression of insoluble aggregating proteins. Proteins expressed at 37 °C visualized on SDS-PAGE gel (**A**). Expression of mTdT at 16 °C visualized on SDS-PAGE gel (**B**). Ins= insoluble fraction, Sol = soluble fraction. The bands marked by arrows indicate the locations of target proteins. Experiment was performed on 3 different expression batches.

Supplementary Table 2: Summary of fusion proteins properties.

Fusion protein	*pI*	*Mw (Da)*	*Activity*
TEV	8.9	26877	Protease
Z_2LO_{10}	5.2	39353	Toxin
M-TdT	6.85	58266	Template independent ssDNA polymerase

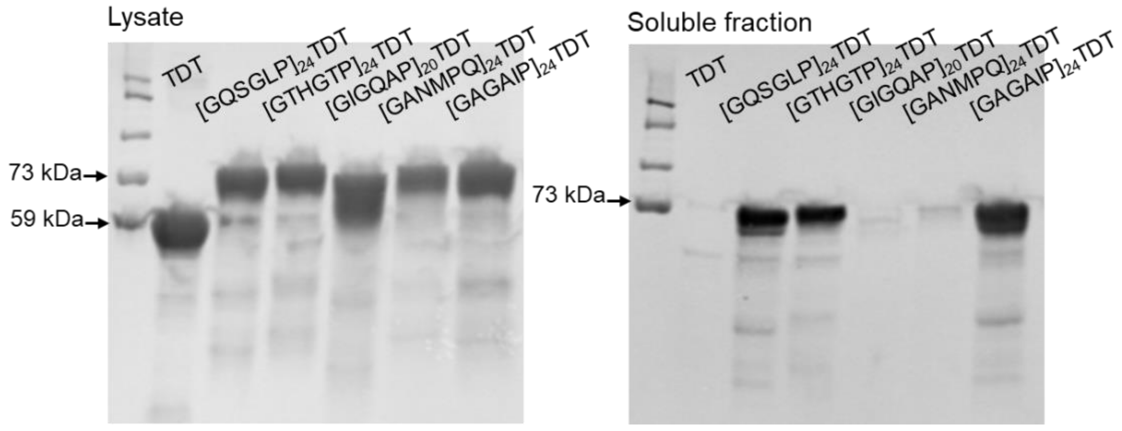

Supplementary Figure 9: Western blots of SynIDPs fused to mTdT. Total cell lysate for each protein (**left**) were separated by centrifugation and the supernatant (**right**) were visualized. Western blots of expressed fusion proteins in lysate were subject to His6-tag antibody detection. Full length fusion proteins are expected to be around 73 kDa in molecular weight, while mTdT is expected to be around 59 kDa in molecular weight. Dark bands (**right**) show soluble expressions of $[GQSGLP]_{24}$-TdT (SynIDP-1-mTdT), $[GTHGTP]_{24}$-TdT (SynIDP-2-mTdT), and $[GAGAIP]_{24}$-TdT (SynIDP-3-mTdT). Experiment was performed on 3 different expression batches.

Supplementary Table 3: Sequences of SynIDPs with theoretical molecular weight and isoelectric points.

IDP	*sequence*	*Mw [Da]*	*pI*	*DNA sequence*
1	$[GQSGLP]_{24}$	15126	5.5	ATGAGCAAAGGACCAGGTCAAAGTGGACTCCCAGGACAATCTGGCTTACCCGGACAGAGCGGTCTTCCAGGGCAATCAGGCTTACCAGGTCAAAGTGGACTCCCAGGACAATCTGGCTTACCCGGACAGAGCGGTCTTCCAGGGCAATCAGGCTTACCAGGTCAAAGTGGACTCCCAGGACAATCTGGCTTACCCGGACAGAGCGGTCTTCCAGGGCAATCAGGCTTACCAGGTCAAAGTGGACTCCCAGGACAATCTGGCTTACCCGGACAGAGCGGTCTTCCAGGGCAATCAGGCTTACCAGGTCAAAGTGGACTCCCAGGACAATCTGGCTTACCCGGACAGAGCGGTCTTCCAGGGCAATCAGGCTTACCAGGTGAAAACCTGTATTTTCAGGGCCATCACCATCACCATCACGGCTAATGATGA
2	$[GTHGTP]_{24}$	15390	6.7	ATGAGCAAAGGACCAGGTACACATGGCACTCCAGGAACTCACGGTACTCCGGGTACGCATGGAACCCCTGGGACTCATGGTACACCAGGTACACATGGCACTCCAGGAACTCACGGTACTCCGGGTACGCATGGAACCCCTGGGACTCATGGTACACCAGGTACACATGGCACTCCAGGAACTCACGGTACTCCGGGTACGCATGGAACCCCTGGGACTCATGGTACACCAGGTACACATGGCACTCCAGGAACTCACGGTACTCCGGGTACGCATGGAACCCCTGGGACTCATGGTACACCAGGTACACATGGCACTCCAGGAACTCACGGTACTCCGGGTACGCATGGAACCCCTGGGACTCATGGTA

				CACCAGGTGAAAACCTGTATTTTCAGGGCCATCAC<br>CATCACCATCACGGCTAATGATGA
3	$[GAGAIP]_{24}$*	1380<br>5	5.5	ATGAGCAAAGGACCAGGTGCCGGTGCAATCCCAG<br>GGGCTGAAGCTATTCCAGGAGCGGGAGCCATACC<br>CGGAGCAGGCGCAATACCAGGTGCCGGTGCAATC<br>CCAGGGGCTGAAGCTATTCCAGGAGCGGGAGCCA<br>TACCCGGAGCAGGCGCAATACCAGGTGCCGGTGC<br>AATCCCAGGGGCTGAAGCTATTCCAGGAGCGGGA<br>GCCATACCCGGAGCAGGCGCAATACCAGGTGCCG<br>GTGCAATCCCAGGGGCTGAAGCTATTCCAGGAGC<br>GGGAGCCATACCCGGAGCAGGCGCAATACCAGGT<br>GCCGGTGCAATCCCAGGGGCTGAAGCTATTCCAG<br>GAGCGGGAGCCATACCCGGAGCAGGCGCAATACC<br>AGGTGCCGGTGCAATCCCAGGGGCTGAAGCTATT<br>CCAGGAGCGGGAGCCATACCCGGAGCAGGCGCAA<br>TACCAGGTGAAAACCTGTATTTTCAGGGCCATCAC<br>CATCACCATCACGGCTAATGATGA

*SynIDP-3 has a missense mutation from G (3rd amino acid) to E, every four repeats.

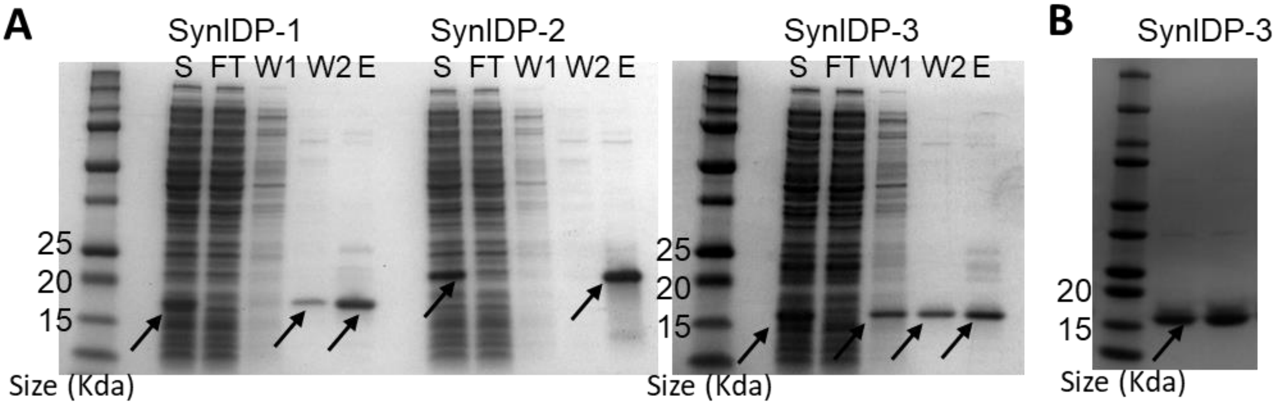

Supplementary Figure 10: Purification of SynIDPs. SynIDP-1/2/3 purification by IMAC (**A**) followed by eluted fraction of SynIDP-3 from SEC (**B**) visualized by SDS-PAGE**.** SynIDPs migrate to a larger molecular weight than expected for globular proteins. S = soluble fraction, FT = flow through, W1 = wash with Lys buffer + 25 mM imidazole, W2 = wash with Lys buffer + 50 mM imidazole, E = elution with Lys buffer + 100 mM imidazole, except for the SynIDP-2 where we used 500 mM imidazole. The bands marked by arrows indicate the locations of target proteins. Experiment was performed on 3 different expression batches

A

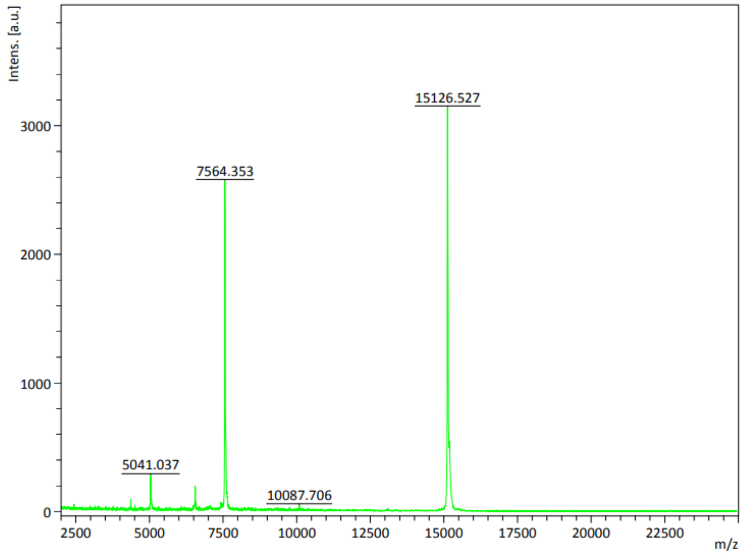

Intens. [a.u.]
3000
2000
1000
0
15126.527
7564.353
5041.037
10087.706
2500
5000
7500
10000
12500
15000
17500
20000
22500
m/z

B

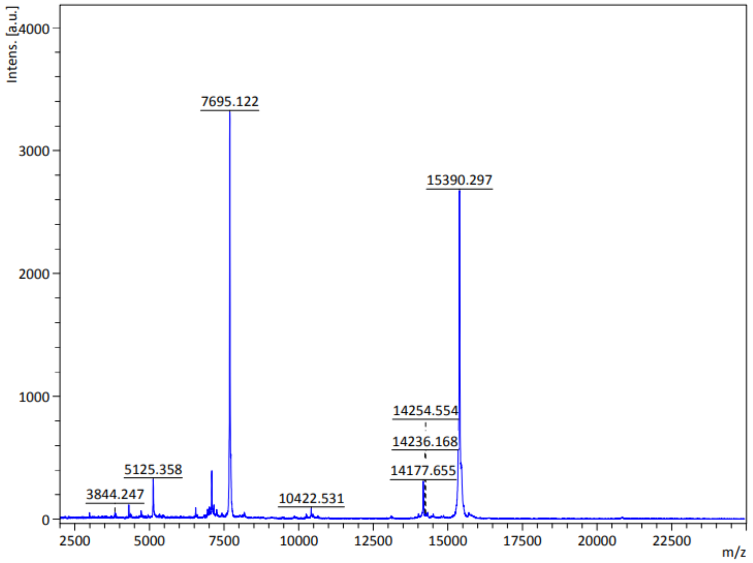

Intens. [a.u.]
4000
3000
2000
1000
0
7695.122
15390.297
14254.554
14236.168
14177.655
5125.358
3844.247
10422.531
2500
5000
7500
10000
12500
15000
17500
20000
22500
m/z

C

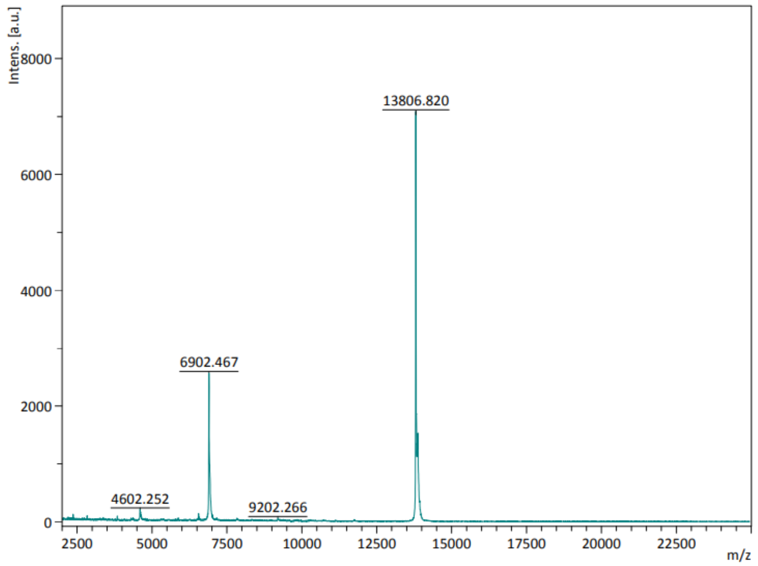

Supplementary Figure 11: MALDI-TOF analysis of SynIDPs. MALDI-TOF spectra confirm SynIDP-1, m/z=15126 **(A),** SynIDP-2, m/z=15390 and smaller truncated product at m/z =14254 **(B),** SynIDP-3, m/z=13806 **(C)** molecular weight. Experiment was performed on 3 different expression batches.

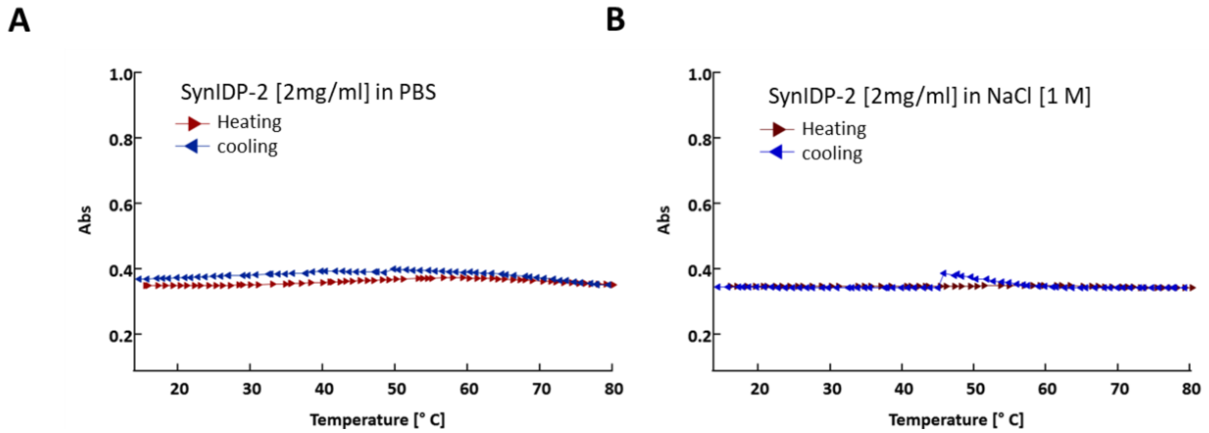

Supplementary Figure 12: Turbidity profiles of SynIDP-2. Optical turbidity of SynIDP-2 in PBS (**A**) and water with 1M NaCl (**B**) was measured at 350 nm with a thermal ramp of 1 °C/min from 15 °C to 80 °C and back to 15 °C. Experiment was performed twice.

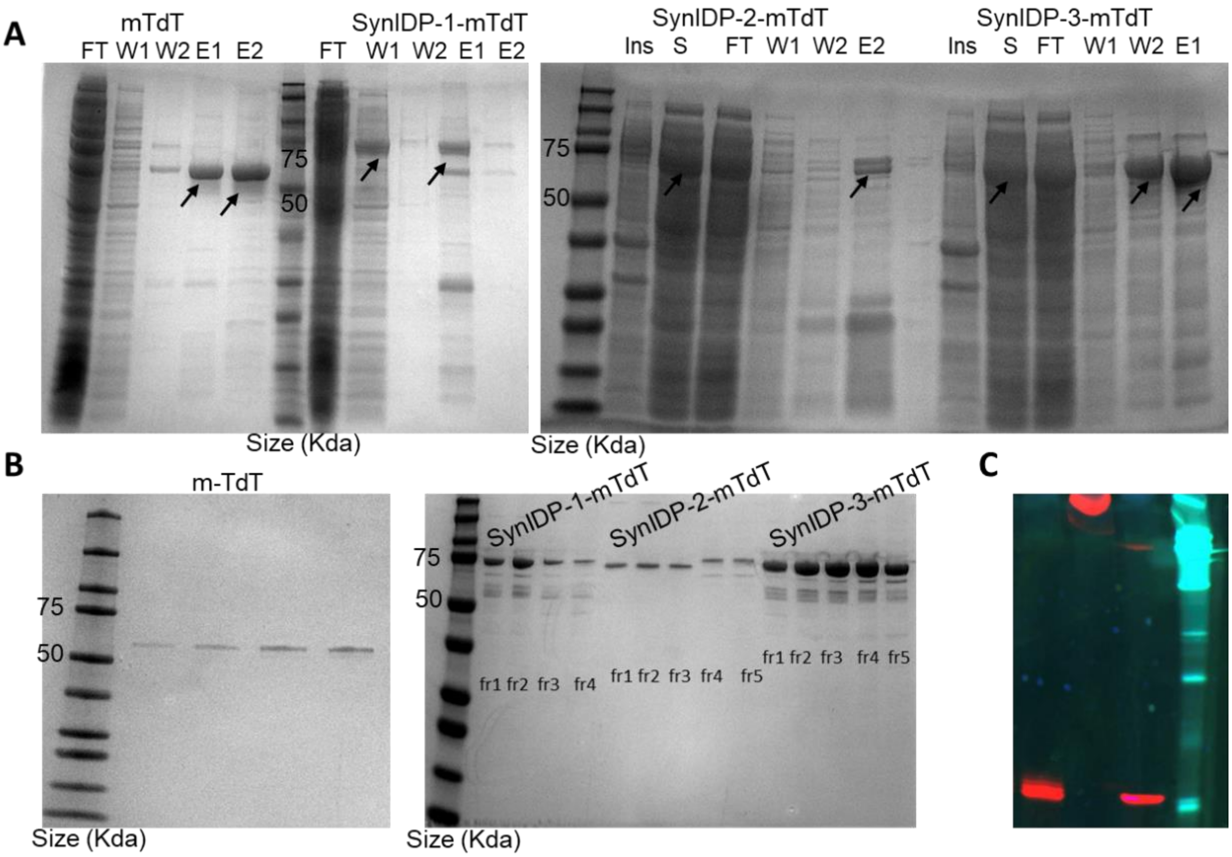

Supplementary Figure 13: Purification of mTdT and SynIDP-mTdT. mTdT and SynIDP-mTdT purification by IMAC (**A**) and eluted fractions from size exclusion (**B**) visualized on SDS-PAGE gel. Ins = insoluble fraction, S = soluble fraction, FT = flow through, W1 = wash with Lys buffer + 25 mM imidazole , W2 = wash with Lys buffer + 50 mM imidazole, E1 = elution with Lys buffer + 100 mM imidazole, E2 = elution with Lys buffer + 500 mM imidazole. The bands marked by arrows indicate the locations of target proteins. SynIDP-2-mTdT fr1-3 (B) were aggregates that eluted prior to fr4-5 and separated under the harsh conditions of SDS-PAGE. However, fr1-3 demonstrated no enzymatic nucleotide addition in vitro activity assays. (**C**) TdT activity assay showing elongation of Cy5-poly-T_{50} initiator on TBE-Urea PAGE gels. Low Range ssRNA ladder was used to quantify nucleotide addition. From left to right; recombinant m-TdT, Promega-TdT (positive control), initiator (negative control), ladder. The assay indicates that recombinant mTdT has very little activity. Experiment was performed on 3 different expression batches.

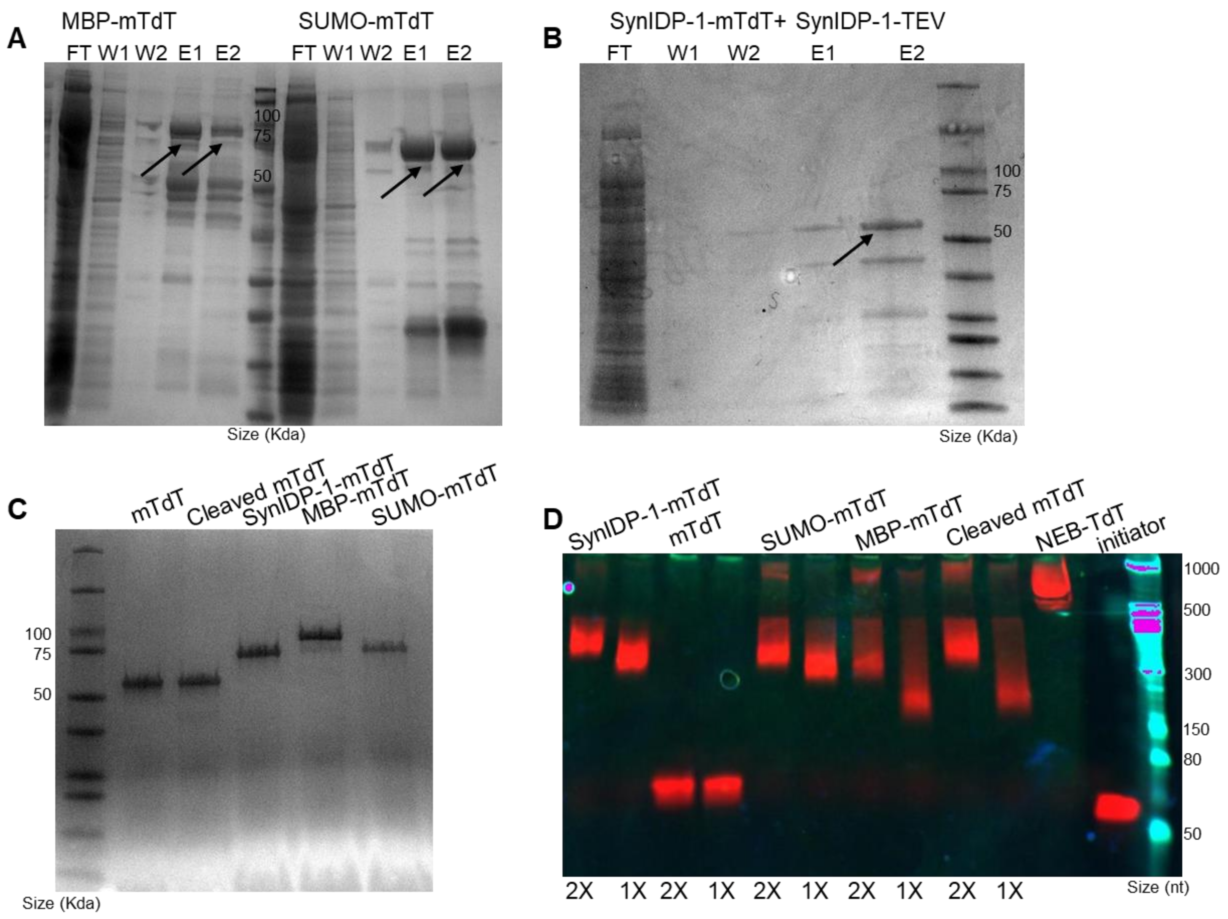

Supplementary Figure 14: Purification of cleaved mTdT, SUMO-mTdT and MBP-TdT. (**A**) MBP-mTdT and SUMO-mTdT purification by IMAC visualized by SDS-PAGE. We note that MBP-His$_6$-mTdT and SUMO-His$_6$-mTdT (no His$_6$ tag at the terminus) were impossible to purify by IMAC (**B**) Soluble fractions from SynIDP-1-mTdT and SynIDP-1-TEV were combined in a 1:1 v:v ratio and incubated at 4 °C overnight. The resulting mixture was purified by IMAC and visualized on SDS-PAGE. The desired products are indicated by arrows. Ins = insoluble fraction, S = soluble fraction, FT = flow through, W1 = wash with Lys buffer + 25 mM imidazole, W2 = wash with Lys buffer + 50 mM imidazole, E1 = elution with Lys buffer + 100 mM imidazole, E2 = elution with Lys buffer + 500 mM imidazole. (**C**) Purified mTdT variants after IMAC and SEC. (**D**) TdT activity assay showing elongation of Cy5-poly-T$_{50}$ initiator on TBE-Urea PAGE gels. Low Range ssRNA ladder was used to quantify nucleotide addition. From left to right; SynIDP-1-mTdT 2X and 1X, recombinant m-TdT 2X and 1X, SUMO-mTdT 2X and 1X, MBP-mTdT 2X and 1X, cleaved mTdT 2X and 1X, NEB-TdT (positive control), initiator (negative control), ladder. The assay indicates that recombinant mTdT has very little activity. In contrast, the cleaved mTdT as well as all the mTdT variants expressed using a solubility tag retain enzymatic activity, with SynIDP-1-mTdT demonstrating the highest processivity and lowest dispersity (Figure 3F). Experiment was performed on 2 different expression batches

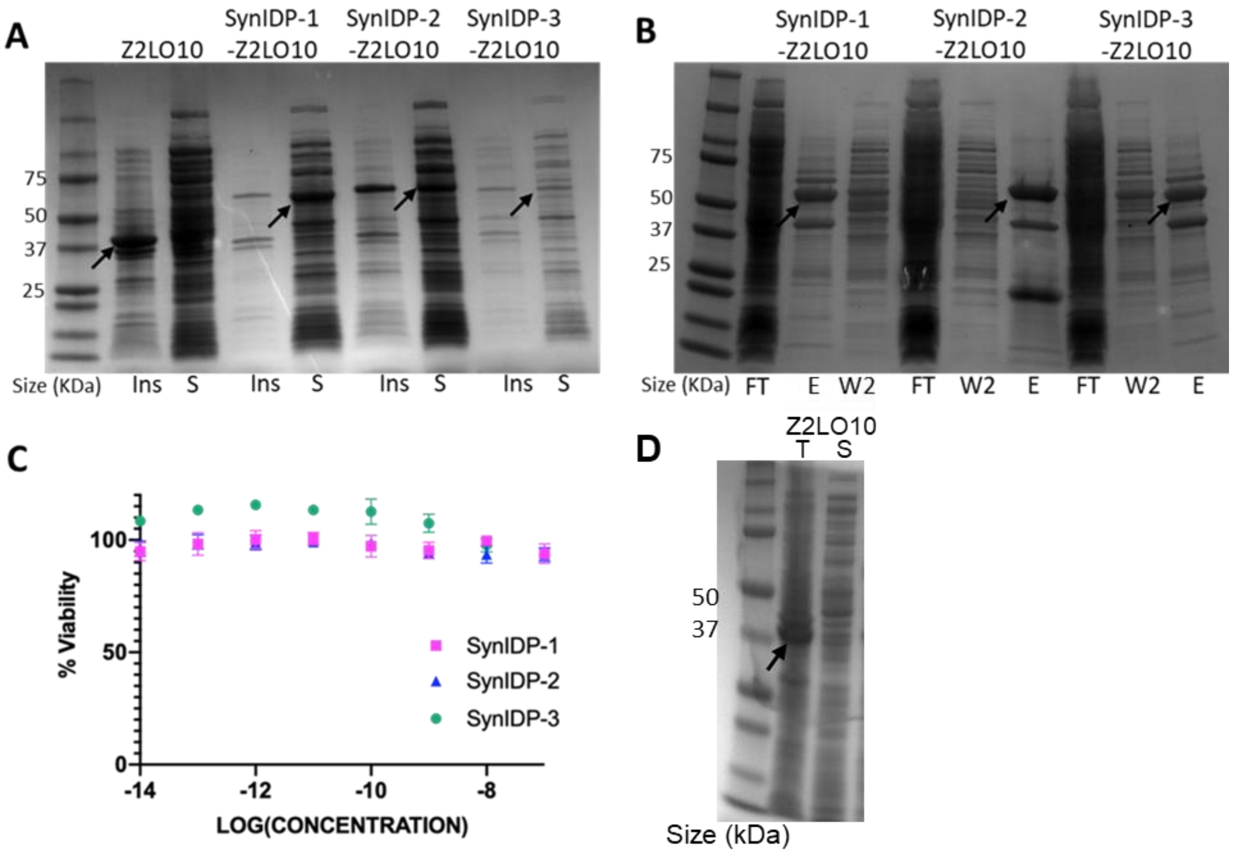

Supplementary Figure 15: Purification and activity of SynIDP-Z2LO10. Z2LO10 and SynIDP-Z2LO10 insoluble and soluble fraction (**A**) and SynIDP-Z2LO10 purification by IMAC (**B**) visualized on SDS-PAGE gel. T=total lysate, Ins = insoluble fraction, S = soluble fraction, FT = flow through, W2 = wash with PBS buffer + 50 mM imidazole, E = elution with PBS buffer + 100 mM imidazole, except for the SynIDP-2-Z2LO10 where we used 500 mM imidazole. (**C**) Log-fold dilutions of SynIDP (controls) were incubated with CT-2A-EGFRviii, an EGFR positive murine glioma cell line for 48 h and tested for viability by an MTS assay. n=3 replicates, error bars represent SD (**D**) Z2LO10 total lysate and soluble fraction visualized on SDS-PAGE gel. The bands marked by arrows indicate the locations of target proteins. Experiment was performed on 2 different expression batches

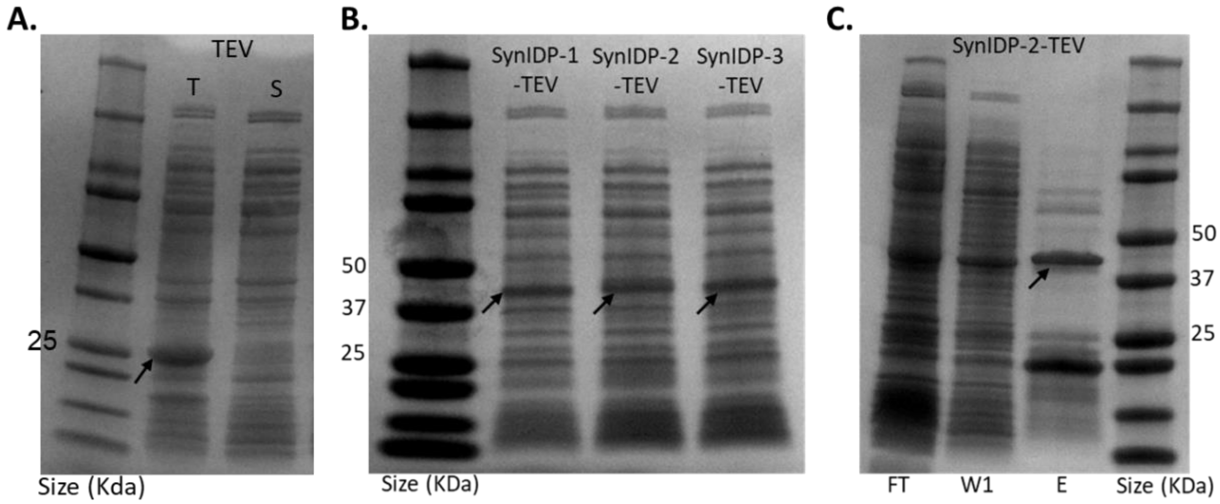

Supplementary Figure 16: **Purification of SynIDP-TEV.** SDS-PAGE visualizes total and soluble expression of TEV alone (**A**) soluble expression of SynIDP-TEV (**B**). Representative SDS-PAGE from IMAC of SynIDP2-TEV (**C**) FT = flow through, W1 = wash with PBS buffer + 50 mM imidazole, E = elution with PBS buffer + 500 mM imidazole. The bands marked by arrows indicate the locations of target proteins. Experiment was performed on 3 different expression batches

GVGVPGVGVPGVGVPGVGVPGVGVPGVGVPGVGVPGVGVPGVGVPGVGVPGVGVPG
VGVPGVGVPGVGVPGVGVPGVGVPGVGVPGVGVPGVGVPGVGVPGVGVPGVGVPGV
GVPGVGVPGVGVPGVGVPGVGVPGVGVPGVGVPGVGVPGVGVPGVGVPGVGVPGVG
VPGVGVPGVGVPGVGVPGVGVPGVGVPGVGVPGVGVPGVGVPGVGVPGVGVPGVGV
PGVGVPGVGVPGVGVPGVGVPGVGVPGVGVPGVGVPGVGVPGVGVPGVGVPGVGVP
GVGVPGVGVPGVGVPGVGVPG*ENLYFQ/G*AYPIPDSSPLLQFGGQVRQRYLYTDDDQDT
EAHLEIREDGTVVGAAHRSPESLLELKALKPGVIQILGVKASRFLCQQPDGALYGSPHFD
PEACSFRERLLEDGYNVYQSEAHGLPLRLPQKDSPNQDATSWGPVRFLPMPGLLHEPQD
QAGFLPPEPPDVGSSDPLSMVEGSQGRSPSYASG

Supplementary Figure 17: ELP-tev-FGF21 substrate amino acid sequence. The red slash indicates the point of cleavage by TEV enzyme.

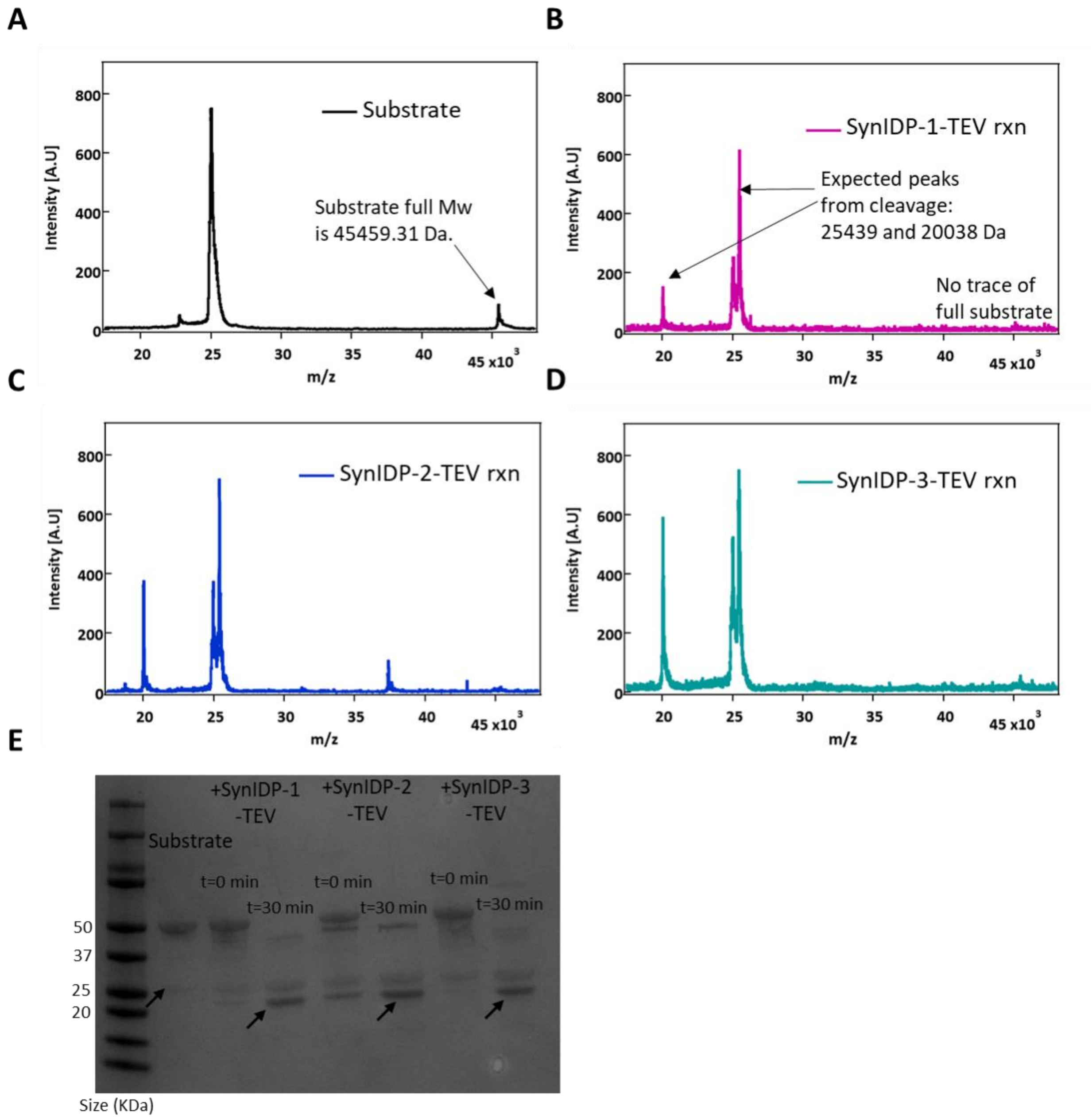

Supplementary Figure 18: MALDI-TOF analysis of products from SynIDP-TEV reaction with ELP-tev-FGF21 substrate. MALDI-TOFMS spectra of negative control-substrate alone show m/z= 45459 as expected, as well as a peak at m/z= 24644 representing the truncated ELP (theoretical Mw=24644 Da) (**A**), substrate incubated for t = 30 min with SynIDP1-TEV (**B**), Syn-IDP2-TEV (**C**) and Syn-IDP3-TEV (**D**). All show appearance of products peaks at expected m/z=25439 and m/z=20038. SDS-PAGE gels visualize tev-containing substrate incubated with SynIDP-TEV for t = 0 min or t = 30 min. The bands marked by arrows indicate the location of the expected cleaved product. Experiment was performed on 2 different expression batches (**E).**

A

Z_2LO_{10}

GGCGTAGATAATAAATTCAATAAGGAGATGTGGGCTGCCTGGGAAGAAATTCGCAA
CTTACCCAATTTAAATGGTTGGCAAATGACCGCTTTCATCGCGAGCCTGGTAGACGA
CCCCTCACAGTCAGCGAATCTGCTGGCTGAGGCGAAAAAGTTAAACGATGCGCAGG
CCCCAAAGGGCGTCGATAATAAATTCAACAAGGAAATGTGGGCCGCCTGGGAGGAA
ATCCGTAACCTTCCTAACTTGAATGGATGGCAGATGACAGCTTTCATCGCGTCTCTT
GTAGACGACCCCTCGCAATCGGCAAATTTACTTGCCGAGGCCAAAAAGTTGAATGA
CGCACAGGCTCCCAAGGGCGGCGGAGGGTCAAAGGCGTCGGGTGGGCGTCATCGTC
AACCGCGCGGCTGGGAACAGTTGGGGGGATCACCAACCGGTGCAGAATTTTTGGGG
GACGGGGGAGATGTTTCGTTTAGCACGCGTGGGACCCAGAATTGGACAGTAGAGCG
CCTTTTGCAAGCGCATCGCCAGTTAGAAGAACGTGGGTATGTGTTCGTCGGGTATCA
CGGAACATTTCTGGAGGCGGCTCAAAGCATCGTTTTTGGCGGAGTACGTGCCCGCAG
TCAGGACCTGGACGCTATCTGGCGTGGATTCTATATTGCTGGAGACCCGGCCTTGGC
ATACGGCTATGCTCAAGATCAAGAGCCCGATGCCGCGGGACGCATTCGCAACGGTG
CGCTGTTACGTGTGTATGTCCCACGTAGTTCCCTTCCAGGTTTTTACCGCACGTCTCT
TACGTTAGCGGCTCCGGAGGCCGCAGGAGAGGTCGAACGTTTGATTGGTCATCCCCT
GCCGCTGCGCTTGGATGCTATCACTGGGCCGGAAGAAGAGGGTGGCCGCTTGGAAA
CGATCTTGGGGTGGCCGCTGGCGGAGCGCACAGTGGTCATCCCTTCGGCAATCCCGA
CAGACCCGCGCAATGTCGGGGGAGACTTGGAACCATCAAGTATTCCAGATAAGGAA
CAGGCGATCTCGGCCCTTCCCGACTATGCCTCTCAACCCGGTAAACCCCCTCGCGAG
GACTTAAAATGATAATAATGATCTTCAGGATCC

B

Primers to linearize the plasmid:

Forward primer:

TGATCTTCAGGATCCGTATTC

GC: 42.9% **Tm:** 51.4ºC

(only 1 stop codon from the plasmid, the rest are deleted)

Reverse primer: GTGATGGTGATGGTGATGGCC

GC: 57.1% **Tm:** 58.5ºC

(His_6, G is deleted)

Primers to exchange the overhangs of the Z_2LO_{10}:

Forward primer: CATCACCATCACCATCACGGCGTAGATAATAAATTCAATAAGGAG

GC: 33.3% **Tm:** 51.7ºC (refers only to the black part which has to anneal)

Reverse primer: TACGGATCCTGAAGATCATTATTATCA

GC: 33.3% **Tm:** 50ºC (refers only to the black part which has to anneal)

Supplementary Figure 19. DNA sequences and primers for Z2-LO10. DNA sequence for Z2-LO10 (**A**). Associated primers used to linearize Syn-IDP containing pET-24a(+) backbone and add correct overhangs to Z2-LO10 genes for Gibson assembly (**B**).
