# Supplementary material for: Synthetic intrinsically disordered protein fusion tags that enhance protein solubility
Source: Nat Commun. 2024 May 2;15:3727. doi: 10.1038/s41467-024-47519-7 (PMC11066018; doi:10.1038/s41467-024-47519-7)
Supplement: Supplementary file 3 — Description of additional supplementary files [file 41467_2024_47519_MOESM3_ESM.pdf]

DESCRIPTION OF ADDITIONAL SUPPLEMENTARY FILES

Supplementary Data 1 : (PGX1X2X3X4)4 motifs encoded by the 1020 microarray-synthesized oligonucleotides.

Supplementary Data 2 : Designed nucleotide sequences of 1020 microarray synthesized oligonucleotides.
