# Supplementary material for: Synthetic intrinsically disordered protein fusion tags that enhance protein solubility
Source: Nat Commun. 2024 May 2;15:3727. doi: 10.1038/s41467-024-47519-7 (PMC11066018; doi:10.1038/s41467-024-47519-7)
Supplement: Supplementary file 6 — Reporting Summary [file 41467_2024_47519_MOESM6_ESM.pdf]

nature portfolio

Corresponding author(s): Ashutosh Chilkoti
Last updated by author(s): 04.03.2024

Reporting Summary

Nature Portfolio wishes to improve the reproducibility of the work that we publish. This form provides structure for consistency and transparency in reporting. For further information on Nature Portfolio policies, see our Editorial Policies and the Editorial Policy Checklist.

Statistics

For all statistical analyses, confirm that the following items are present in the figure legend, table legend, main text, or Methods section.

n/a	Confirmed	
☐	☒	The exact sample size (*n*) for each experimental group/condition, given as a discrete number and unit of measurement
☐	☒	A statement on whether measurements were taken from distinct samples or whether the same sample was measured repeatedly
☒	☐	The statistical test(s) used AND whether they are one- or two-sided<br>*Only common tests should be described solely by name; describe more complex techniques in the Methods section.*
☐	☒	A description of all covariates tested
☐	☒	A description of any assumptions or corrections, such as tests of normality and adjustment for multiple comparisons
☐	☒	A full description of the statistical parameters including central tendency (e.g. means) or other basic estimates (e.g. regression coefficient) AND variation (e.g. standard deviation) or associated estimates of uncertainty (e.g. confidence intervals)
☒	☐	For null hypothesis testing, the test statistic (e.g. *F*, *t*, *r*) with confidence intervals, effect sizes, degrees of freedom and *P* value noted<br>*Give P values as exact values whenever suitable.*
☒	☐	For Bayesian analysis, information on the choice of priors and Markov chain Monte Carlo settings
☐	☒	For hierarchical and complex designs, identification of the appropriate level for tests and full reporting of outcomes
☒	☐	Estimates of effect sizes (e.g. Cohen's *d*, Pearson's *r*), indicating how they were calculated

Our web collection on statistics for biologists contains articles on many of the points above.

Software and code

Policy information about availability of computer code

Data collection	All data were collected using the commercial programs provided with the instruments used. Image Lab was used for imaging SDS Protein gels. Amersham Imager 600 was used to image western blots. flexAnalysis was used for MALDI-TOF MS. SAXSGUI was used for collecting SAXS data.
Data analysis	During data analysis, image thresholding was performed in ImageJ to identify colonies containing soluble protein.the Burrows-wheeler aligner (BWA) was used for mapping sequences generated from Illumina Miseq against the 1020 reference sequences which were used for oligonucleotide synthesis. IgorPro and SASVIEW were used for SAXS analysis. ImageJ was used for mTdT activity assays.

For manuscripts utilizing custom algorithms or software that are central to the research but not yet described in published literature, software must be made available to editors and reviewers. We strongly encourage code deposition in a community repository (e.g. GitHub). See the Nature Portfolio guidelines for submitting code & software for further information.

Data

Policy information about availability of data

All manuscripts must include a data availability statement. This statement should provide the following information, where applicable:

- Accession codes, unique identifiers, or web links for publicly available datasets
- A description of any restrictions on data availability
- For clinical datasets or third party data, please ensure that the statement adheres to our policy

Source data are provided with this paper. The datasets generated during and/or analyzed during the current study are available in the Figshare repository (https://doi.org/10.6084/m9.figshare.25351921)99. Sequencing data for SynIDPs can be found through GenBank accession numbers (PP454715 [https://www.ncbi.nlm.nih.gov/nuccore/PP454715.1], PP454716 [https://www.ncbi.nlm.nih.gov/nuccore/PP454716.1], and PP454717 [https://www.ncbi.nlm.nih.gov/nuccore/PP454717.1]). NGS Data has been deposited to NCBI Sequence Read Archive and can be found with accession numbers (SAMN40567615 [https://www.ncbi.nlm.nih.gov/biosample/?term=SAMN40567615], and SAMN40567616 [https://www.ncbi.nlm.nih.gov/biosample/?term=SAMN40567616]).

Research involving human participants, their data, or biological material

Policy information about studies with human participants or human data. See also policy information about sex, gender (identity/presentation), and sexual orientation and race, ethnicity and racism.

Reporting on sex and gender	N/A
Reporting on race, ethnicity, or other socially relevant groupings	N/A
Population characteristics	N/A
Recruitment	N/A
Ethics oversight	N/A

Note that full information on the approval of the study protocol must also be provided in the manuscript.

Field-specific reporting

Please select the one below that is the best fit for your research. If you are not sure, read the appropriate sections before making your selection.

☒ Life sciences ☐ Behavioural & social sciences ☐ Ecological, evolutionary & environmental sciences

For a reference copy of the document with all sections, see nature.com/documents/nr-reporting-summary-flat.pdf

Life sciences study design

All studies must disclose on these points even when the disclosure is negative.

Sample size	Sample sizes were determined by the least number of replicates needed to perform necessary statistics. In most cases, triplicates were performed for activity of enzymes or proteins. Target proteins were expressed at least three times and every batch was tested to confirm similar activity. Data with only one replicate is representative of results seen throughout each batch's testing.
Data exclusions	No data was exluded
Replication	At least three batches of the same protein were expressed at different times, and at each instance, the Identity of proteins were tested and characterized prior to each test. Results shown in manuscript were confirmed at least 2 separate occasions, or in triplicate testing.
Randomization	*Describe how samples/organisms/participants were allocated into experimental groups. If allocation was not random, describe how covariates were controlled OR if this is not relevant to your study, explain why.*
Blinding	Blinding was not performed in this study

Reporting for specific materials, systems and methods

We require information from authors about some types of materials, experimental systems and methods used in many studies. Here, indicate whether each material, system or method listed is relevant to your study. If you are not sure if a list item applies to your research, read the appropriate section before selecting a response.

Materials & experimental systems

n/a	Involved in the study
☐	☒ Antibodies
☐	☒ Eukaryotic cell lines
☒	☐ Palaeontology and archaeology
☒	☐ Animals and other organisms
☒	☐ Clinical data
☒	☐ Dual use research of concern
☒	☐ Plants

Methods

n/a	Involved in the study
☒	☐ ChIP-seq
☒	☐ Flow cytometry
☒	☐ MRI-based neuroimaging

Antibodies

Antibodies used: 1:2000 dilution of Anti-TdT Mouse Monoclonal Antibody [7H9A33]; 1:4000 dilution of DyLight 650 conjugated 6x-His epitope tag antibody (Life Technologies, cat. no. MA1-21315-D650)

Validation: Each lot of this antibody is quality control tested by Western blotting.

Eukaryotic cell lines

Policy information about cell lines and Sex and Gender in Research

Cell line source(s): CT-2A Glioma lines transfected with EGFRvIII (A gift from the Bigner Lab, Duke University)

Authentication: Cells were authenticated via flow cytometry prior to use in a previous study

Mycoplasma contamination: Cells were not tested for mycoplasma

Commonly misidentified lines (See ICLAC register): *Name any commonly misidentified cell lines used in the study and provide a rationale for their use.*
