# Supplementary material for: Synthetic intrinsically disordered protein fusion tags that enhance protein solubility
Source: Nat Commun. 2024 May 2;15:3727. doi: 10.1038/s41467-024-47519-7 (PMC11066018; doi:10.1038/s41467-024-47519-7)
Supplement: Supplementary file 7 — Source Files [file 41467_2024_47519_MOESM7_ESM.zip › source files/MSdata- Figure 4 S11 S18/1Z2LO10.pdf]

D:\Data\2022\Therien\Francesco\sbinol_tegOH\6M_60min\0_B9\1\1SLin

Acquisition Parameter

Date of acquisition	2022-04-25T15:04:45.899-04:00
Acquisition method name	D:\Methods\flexControlMethods\LP_4-25_kDa.par
Aquisition operation mode	Linear
Voltage polarity	POS
Number of shots	2000
Name of spectrum used for calibration	
Calibration reference list used	Protein1CalibStandard_+aldolase

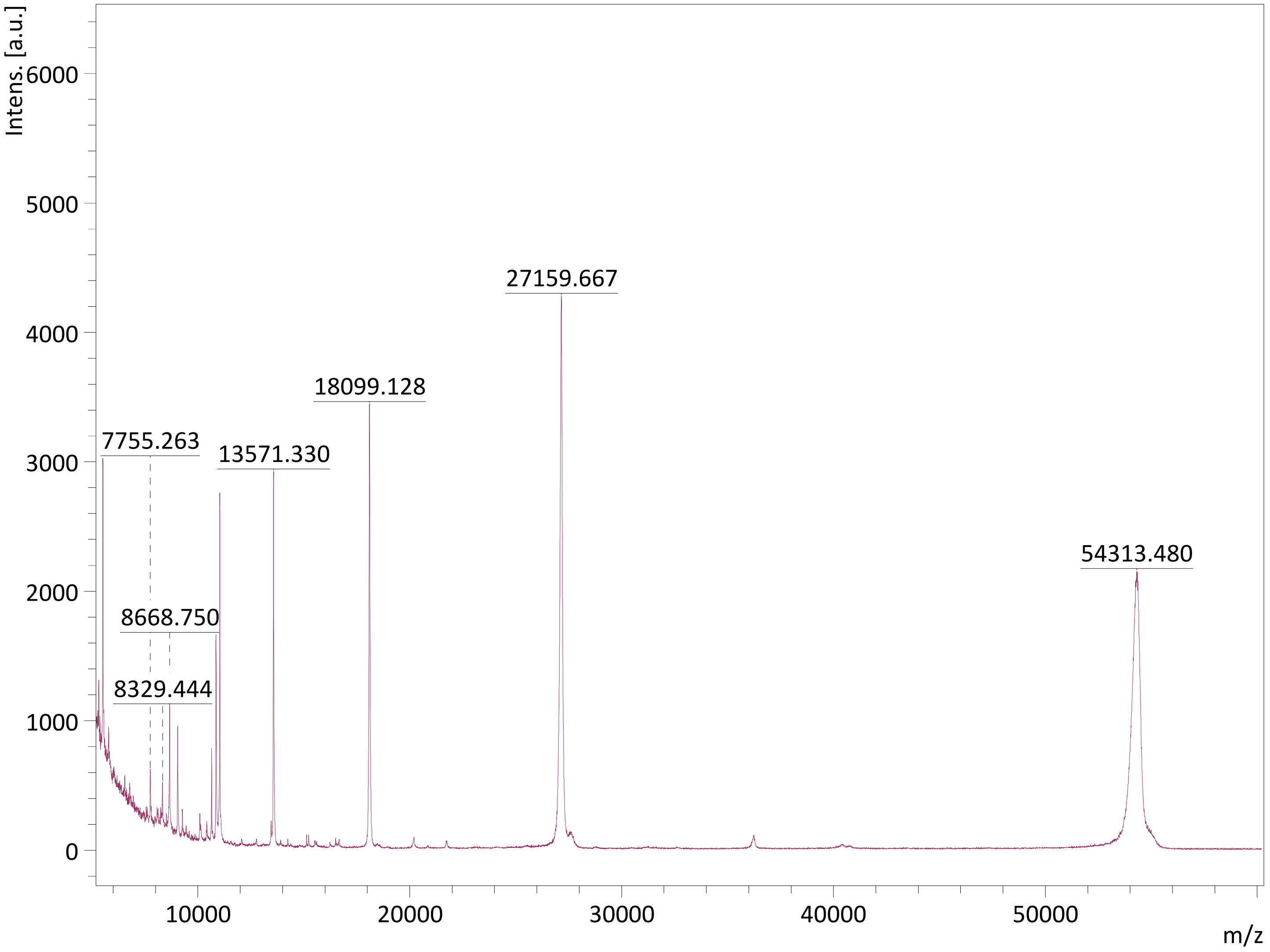

Mass List

m/z	Intens.
5205.284	1018
5209.421	1033
5216.676	1026
5230.491	1014
5235.229	1027
5269.660	1061
5275.901	1075

m/z	Intens.
5292.596	1037
5322.062	1307
5326.148	1279
5378.532	1032
5381.679	1021
5501.638	1135
5502.460	1140
5516.964	3028
5520.140	2954
5539.798	1108
5545.008	1078
5553.750	1084
5554.606	1060
5555.692	1073
5789.093	934
5793.097	938
5796.000	897
7751.175	612
7755.263	628
7766.641	465
8329.444	531
8636.342	430
8637.565	418
8646.900	514
8652.437	557
8668.750	1106
9047.055	939
9047.218	911
9065.520	469
9071.757	409
9077.310	349
10650.210	789
10856.583	1659
10997.960	379
11036.796	2759
11066.549	296
13571.330	2925
18099.128	3447
26935.614	257
26948.947	281
26985.123	475
27148.958	4214
27159.667	4281
53724.238	253
53748.718	250
53748.718	250
53773.344	281
53793.452	310
53809.148	326
53809.148	326
53830.300	369
53849.160	388
53870.543	422
53894.009	474
53905.376	499
53931.982	565
53934.257	566
53954.391	638
53967.804	665
54007.984	818
54025.118	859
54038.306	962
54056.401	999

m/z	Intens.
54102.024	1230
54115.557	1307
54149.129	1488
54185.747	1651
54197.289	1706
54229.879	1902
54232.828	1902
54260.079	2080
54273.627	2083
54291.523	2064
54313.480	2157
54331.134	2094
54334.498	2088
54337.584	2135
54340.951	2122
54344.315	2148
54351.053	2108
54377.989	2078
54391.467	1968
54405.991	1852
54428.767	1659
54476.255	1275
54533.277	795
54644.440	323
54661.334	316
54672.919	298
54699.622	261
54701.880	260
54705.259	260
54708.638	259
