# Supplementary material for: Synthetic intrinsically disordered protein fusion tags that enhance protein solubility
Source: Nat Commun. 2024 May 2;15:3727. doi: 10.1038/s41467-024-47519-7 (PMC11066018; doi:10.1038/s41467-024-47519-7)
Supplement: Supplementary file 7 — Source Files [file 41467_2024_47519_MOESM7_ESM.zip › source files/MSdata- Figure 4 S11 S18/Figure S11/SynIDP1.pdf]

D:\Data\2022\Chilkoti\Yulia\03.01.22\13_1to10\0_G4\1\1SLin

Acquisition Parameter

Date of acquisition	2022-03-01T13:38:23.694-05:00
Acquisition method name	D:\Methods\flexControlMethods\LP_4-25_kDa.par
Aquisition operation mode	Linear
Voltage polarity	POS
Number of shots	1500
Name of spectrum used for calibration	
Calibration reference list used	Protein1CalibStandard

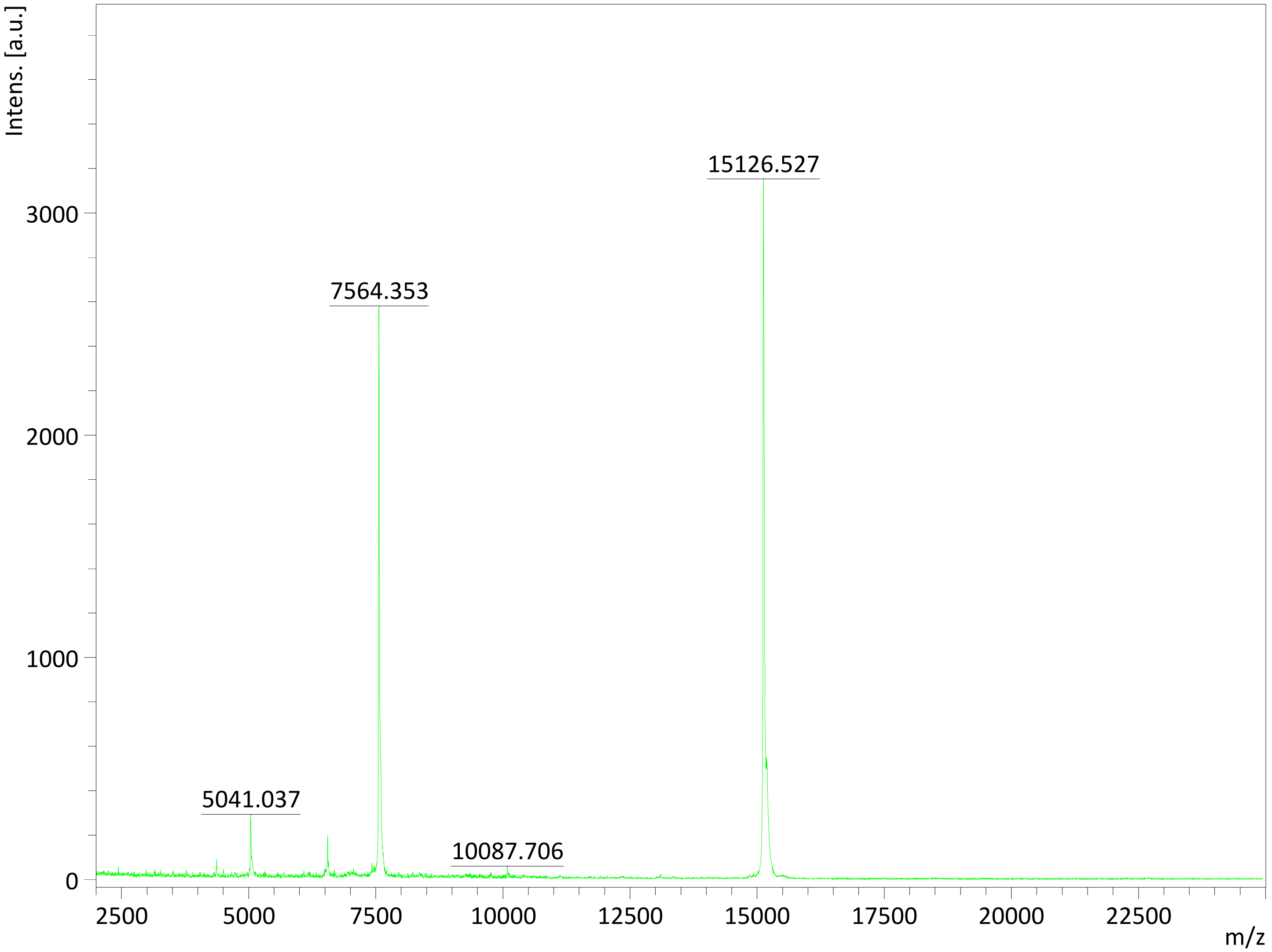

Mass List

m/z	Intens.
2441.948	56.0
4371.960	93.0
5020.050	53.0
5041.037	282
5063.750	104
5071.159	87.0
5085.029	55.0

D:\Data\2022\Chilkoti\Yulia\03.01.22\13_1to10\0_G4\1\1SLin

m/z	Intens.
6506.021	49.0
6554.563	199
6571.608	82.0
7424.936	73.0
7479.608	59.0
7488.123	53.0
7509.408	57.0
7518.788	72.0
7564.353	2570
7599.616	529
7632.411	151
7663.805	73.0
7677.638	52.0
10087.706	47.0
15018.492	38.0
15048.460	41.0
15126.527	3141
15183.748	547
15194.077	533
15305.667	60.0
15320.001	43.0
15335.057	32.0
