# Supplementary material for: Synthetic intrinsically disordered protein fusion tags that enhance protein solubility
Source: Nat Commun. 2024 May 2;15:3727. doi: 10.1038/s41467-024-47519-7 (PMC11066018; doi:10.1038/s41467-024-47519-7)
Supplement: Supplementary file 7 — Source Files [file 41467_2024_47519_MOESM7_ESM.zip › source files/MSdata- Figure 4 S11 S18/Figure S11/SynIDP2.pdf]

Acquisition Parameter

Date of acquisition	2022-03-01T13:43:17.229-05:00
Acquisition method name	D:\Methods\flexControlMethods\LP_4-25_kDa.par
Aquisition operation mode	Linear
Voltage polarity	POS
Number of shots	2500
Name of spectrum used for calibration	
Calibration reference list used	Protein1CalibStandard

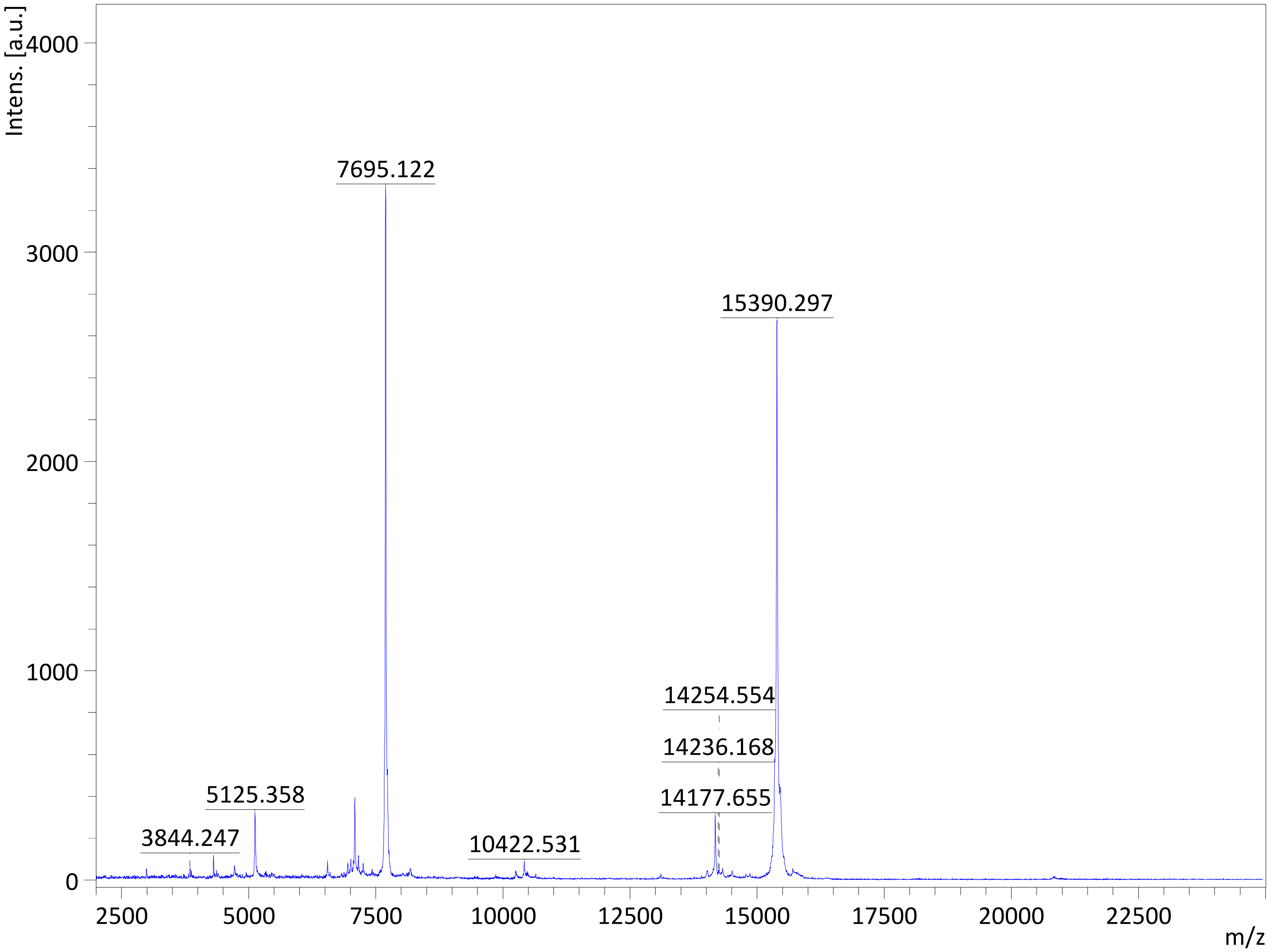

Mass List

m/z	Intens.
2993.166	54.0
3844.247	55.0
3851.407	53.0
3854.592	52.0
3872.952	44.0
4311.546	107
4369.337	47.0

m/z	Intens.
4723.930	65.0
4732.955	47.0
5125.358	324
5143.907	120
5149.180	70.0
5157.884	56.0
5161.869	62.0
5171.195	49.0
6552.663	93.0
6946.411	64.0
6950.174	82.0
6955.846	72.0
7004.151	80.0
7011.365	101
7018.997	59.0
7043.558	60.0
7061.246	91.0
7068.673	76.0
7070.002	85.0
7088.750	395
7111.093	82.0
7132.097	59.0
7138.307	62.0
7159.643	117
7170.567	66.0
7253.814	79.0
7627.117	65.0
7695.122	3313
7729.946	529
7757.256	142
7765.467	128
7790.681	54.0
8179.930	56.0
10251.722	44.0
10422.531	88.0
10486.944	40.0
14016.041	44.0
14026.756	43.0
14139.232	55.0
14177.655	310
14236.168	46.0
14247.498	45.0
14254.554	38.0
14322.892	56.0
14510.540	43.0
15257.954	52.0
15271.740	82.0
15289.966	116
15349.153	579
15390.297	2674
15448.829	416
15456.152	446
15525.735	116
15568.672	44.0
15586.623	39.0
15705.364	42.0
15713.775	39.0
15733.017	38.0
15733.017	38.0
15754.076	37.0
