# Supplementary material for: Synthetic intrinsically disordered protein fusion tags that enhance protein solubility
Source: Nat Commun. 2024 May 2;15:3727. doi: 10.1038/s41467-024-47519-7 (PMC11066018; doi:10.1038/s41467-024-47519-7)
Supplement: Supplementary file 7 — Source Files [file 41467_2024_47519_MOESM7_ESM.zip › source files/MSdata- Figure 4 S11 S18/Figure S11/SynIDP3.pdf]

D:\Data\2022\Chilkoti\Yulia\03.01.22\47_1to10\0_G10\1\1SLin

Acquisition Parameter

Date of acquisition	2022-03-01T13:45:16.442-05:00
Acquisition method name	D:\Methods\flexControlMethods\LP_4-25_kDa.par
Aquisition operation mode	Linear
Voltage polarity	POS
Number of shots	2500
Name of spectrum used for calibration	
Calibration reference list used	Protein1CalibStandard

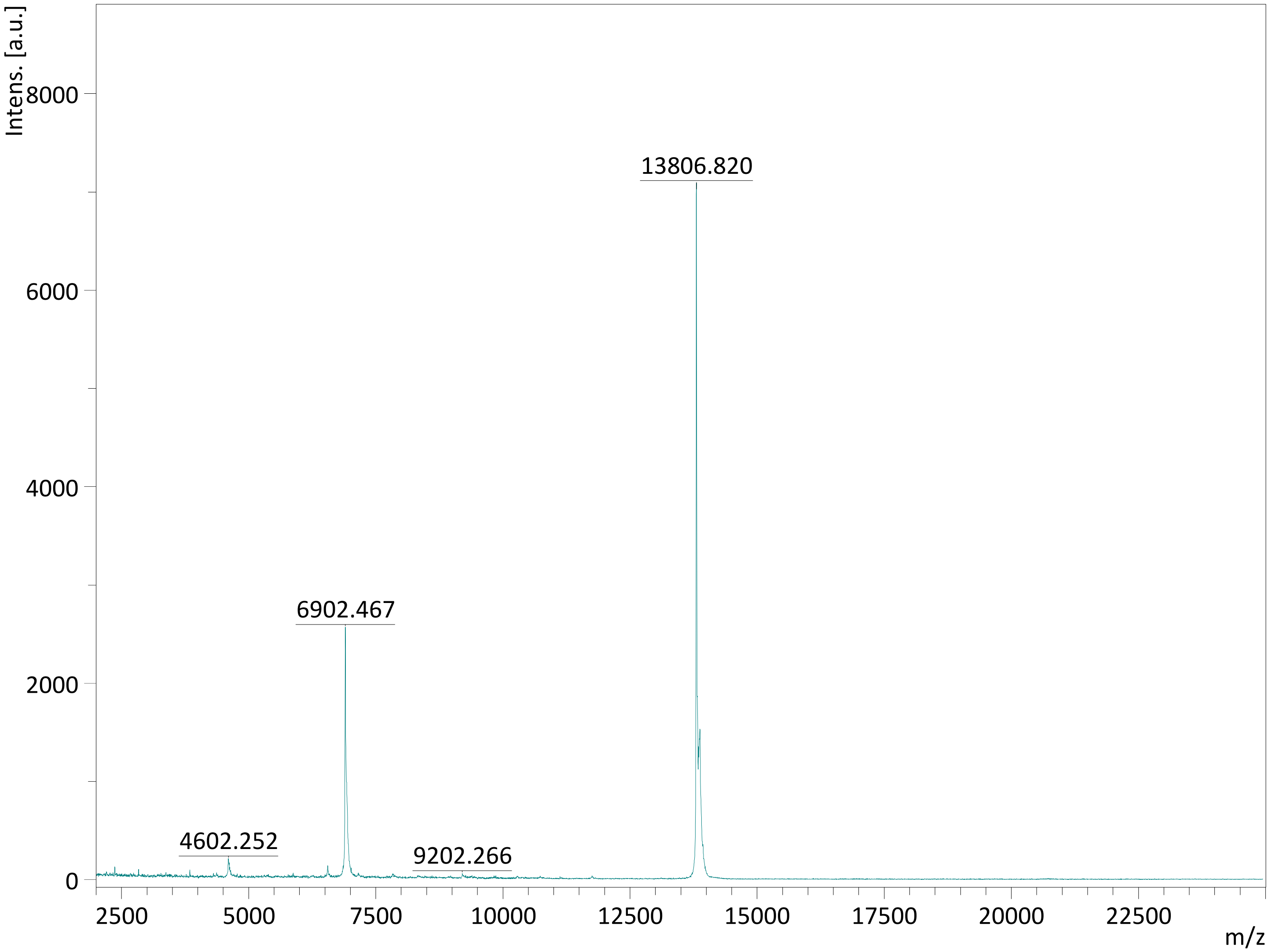

Mass List

m/z	Intens.
2368.394	130
2838.083	105
3844.495	100
4602.252	213
4607.424	158
4615.438	156
4617.621	170

m/z	Intens.
4627.882	120
4642.040	81.0
4648.542	92.0
6555.870	141
6844.426	78.0
6856.110	134
6862.685	145
6902.467	2570
6926.004	994
6981.049	188
7004.150	105
7018.580	120
9202.266	65.0
13684.879	39.0
13708.438	53.0
13722.471	70.0
13806.820	7019
13824.390	1869
13849.081	1353
13875.262	1532
13934.694	335
13979.119	116
13990.094	95.0
14016.773	57.0
14034.809	42.0
