# Supplementary material for: Synthetic intrinsically disordered protein fusion tags that enhance protein solubility
Source: Nat Commun. 2024 May 2;15:3727. doi: 10.1038/s41467-024-47519-7 (PMC11066018; doi:10.1038/s41467-024-47519-7)
Supplement: Supplementary file 7 — Source Files [file 41467_2024_47519_MOESM7_ESM.zip › source files/MSdata- Figure 4 S11 S18/Figure S18/SynIDP1TEV_RXN.pdf]

Acquisition Parameter

Date of acquisition	2023-02-13T11:42:49.083-05:00
Acquisition method name	D:\Data\2023\Chilkoti\Josh\Methods\LP_10-50_Aldolase_Calibration.par
Aquisition operation mode	Linear
Voltage polarity	POS
Number of shots	7000
Name of spectrum used for calibration	
Calibration reference list used	Protein1CalibStandard_+aldolase_+albumin

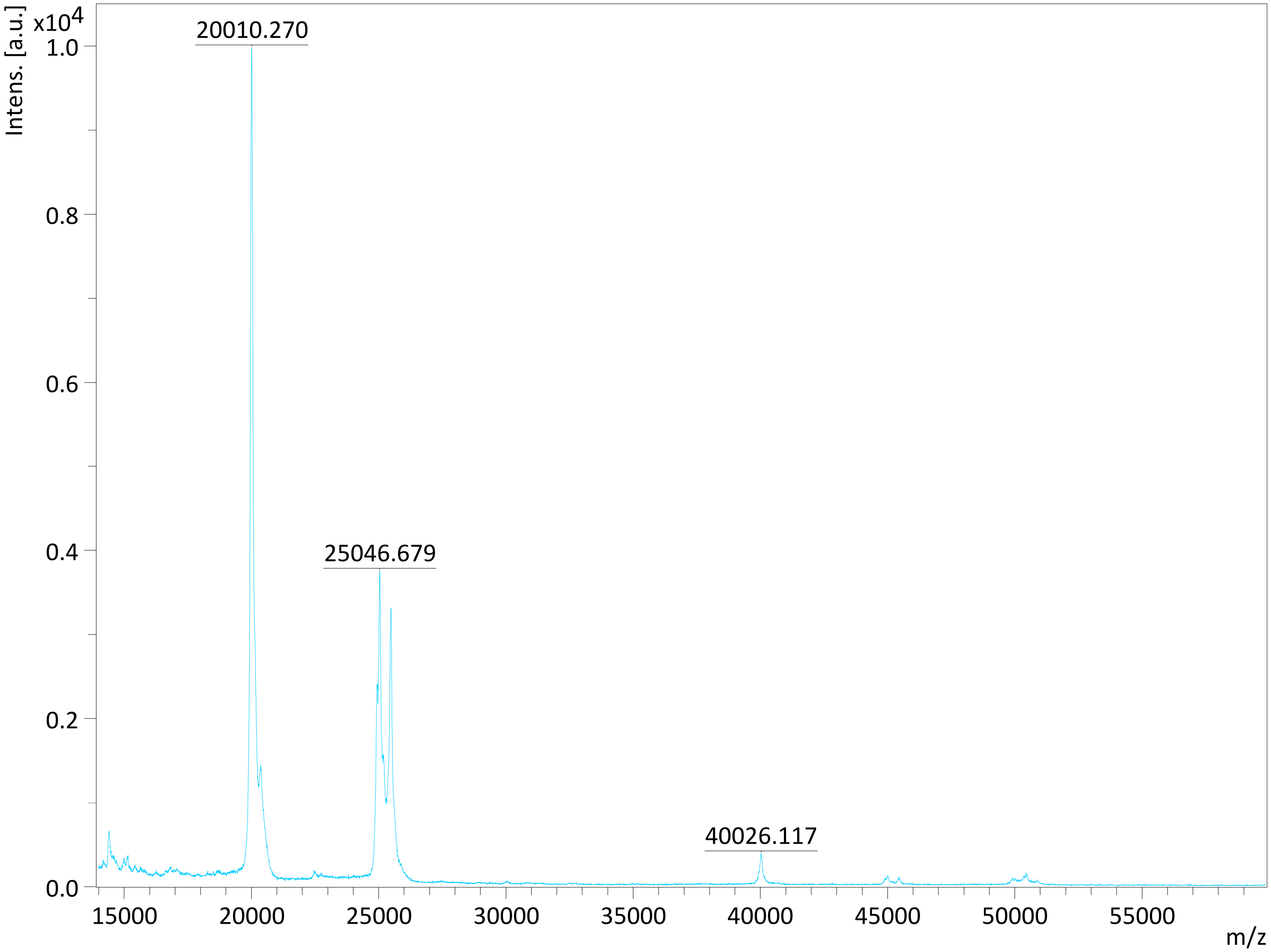

Mass List

m/z	Intens.
14374.011	508
14382.983	570
14405.245	657
14414.124	666
14423.695	547
14432.934	555
14442.175	525

m/z	Intens.
14453.735	485
14464.908	451
19761.509	389
19765.625	391
19781.901	439
19796.781	502
19809.655	552
19833.794	702
20010.270	9983
20133.739	2978
20176.626	2296
20231.618	1440
20246.950	1314
20259.768	1257
20276.388	1221
20291.459	1148
20301.728	1214
20324.760	1291
20331.836	1317
20339.271	1366
20364.741	1444
20377.407	1376
20386.390	1400
20386.589	1363
20406.254	1225
20423.106	1086
20435.715	1049
20443.285	1027
20454.000	957
20462.270	917
20473.254	920
20480.606	868
20503.043	777
20506.427	767
20534.390	693
20539.009	699
20546.911	647
20565.146	603
20593.751	517
20609.797	500
20623.914	443
20625.500	426
20641.002	423
20656.097	371
24805.176	377
24825.631	515
24924.771	2274
24938.056	2403
24947.182	2374
24958.922	2371
24980.567	2290
25038.676	3670
25046.679	3759
25120.863	1716
25143.359	1532
25150.253	1557
25157.100	1556
25182.271	1562
25189.472	1563
25191.426	1572
25205.327	1506
25212.000	1465
25219.148	1437

D:\Data\2023\Chilkoti\Yulia\IDP.TEV02.13\13TEV hcca 14to60kDa\0_B7\1\1SLin

m/z	Intens.
25255.538	1144
25274.114	1018
25286.866	994
25305.475	1055
25322.229	1078
25333.506	1101
25335.462	1112
25357.770	1228
25372.512	1344
25381.690	1384
25409.897	1606
25482.009	3315
25565.993	1212
25577.333	1108
25584.243	1072
25609.181	978
25623.747	919
25634.230	836
25641.218	820
25681.179	601
25727.414	438
25733.799	404
25745.158	387
25756.055	373
25766.725	360
25784.497	323
39988.517	266
40026.117	396
40033.122	388
40055.122	346
