# Supplementary material for: Synthetic intrinsically disordered protein fusion tags that enhance protein solubility
Source: Nat Commun. 2024 May 2;15:3727. doi: 10.1038/s41467-024-47519-7 (PMC11066018; doi:10.1038/s41467-024-47519-7)
Supplement: Supplementary file 7 — Source Files [file 41467_2024_47519_MOESM7_ESM.zip › source files/MSdata- Figure 4 S11 S18/Figure S18/SynIDP2TEV_RXN.pdf]

C:\Users\ys362\Desktop\Yulia MS\Yulia
MS\IDP.TEV02.13\24TEVrxn\0_B2\1\1SLin

Comment 1

Comment 2

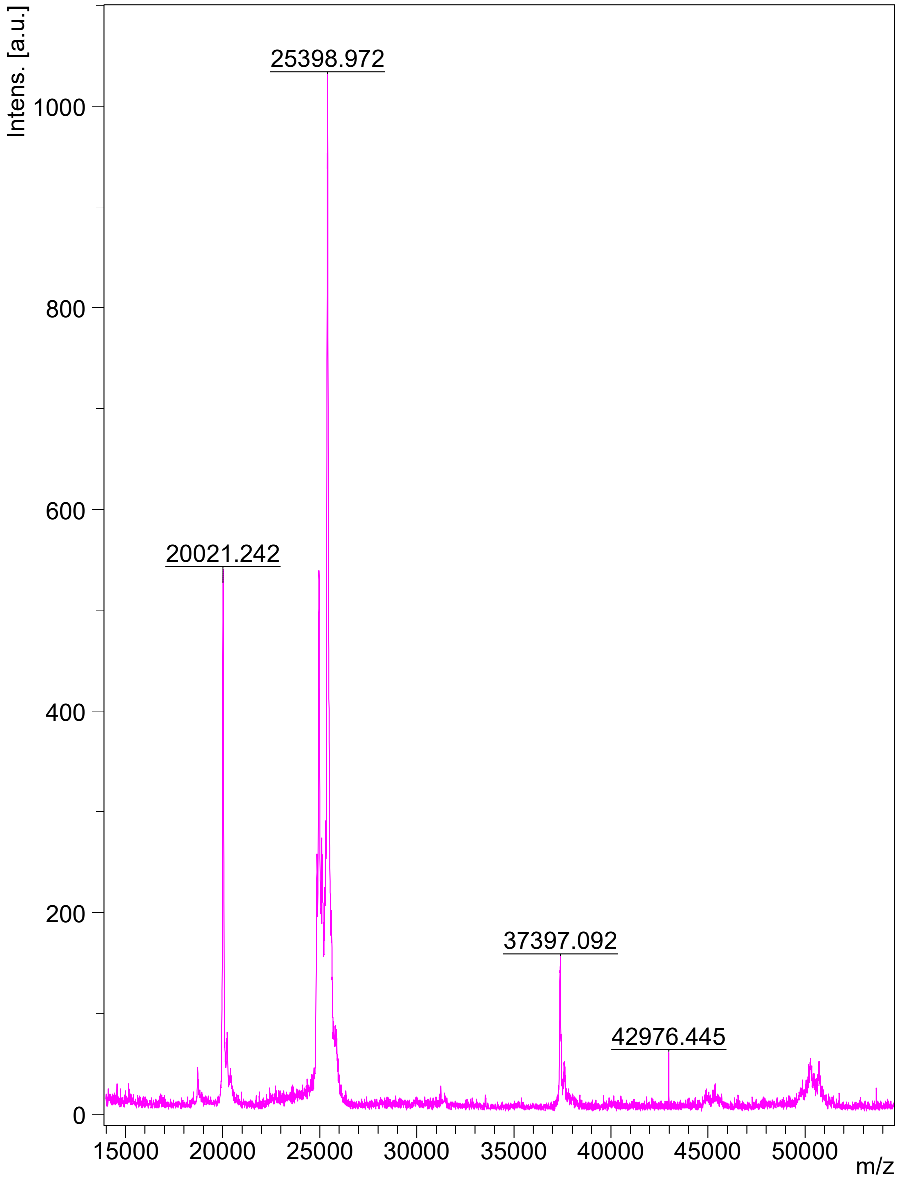

Acquisition Parameter

Date of acquisition	2023-02-21T13:12:33.696-05:00
Acquisition method name	D:\Methods\flexControlMethods\LP_20-50_kDa.par
Spectrum type	TOF
Parent ion mass for ms/ms spectra	
Parent mass + PCIS upper mass limit	
Parent mass - PCIS lower mass limit	
CID valve mode	

Instrument Info

User	FLEX-USER
Instrument	ATS-00715
Instrument type	autoflex
