# Supplementary figures and images for: Synthetic intrinsically disordered protein fusion tags that enhance protein solubility
Source: Nat Commun. 2024 May 2;15:3727. doi: 10.1038/s41467-024-47519-7 (PMC11066018; doi:10.1038/s41467-024-47519-7)

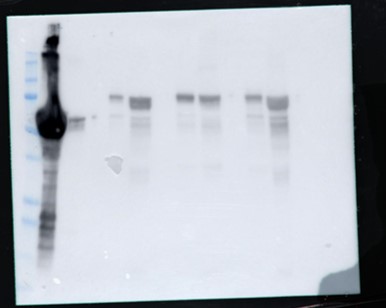

Supplement: Supplementary file 7 — Source Files [file 41467_2024_47519_MOESM7_ESM.zip › source files/western blot- Figure 3A/anti-TdT.jpg]

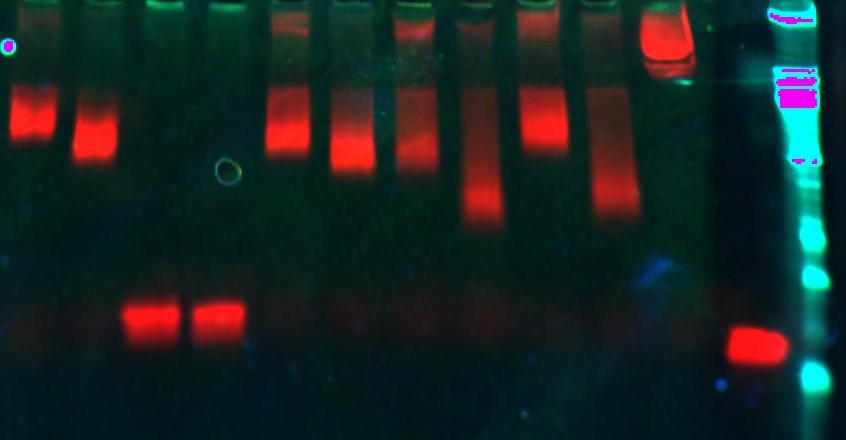

Supplement: Supplementary file 7 — Source Files [file 41467_2024_47519_MOESM7_ESM.zip › source files/TdT ASSAY-Figure 3/2023.12.02_21.58.49_Fl.jpg]

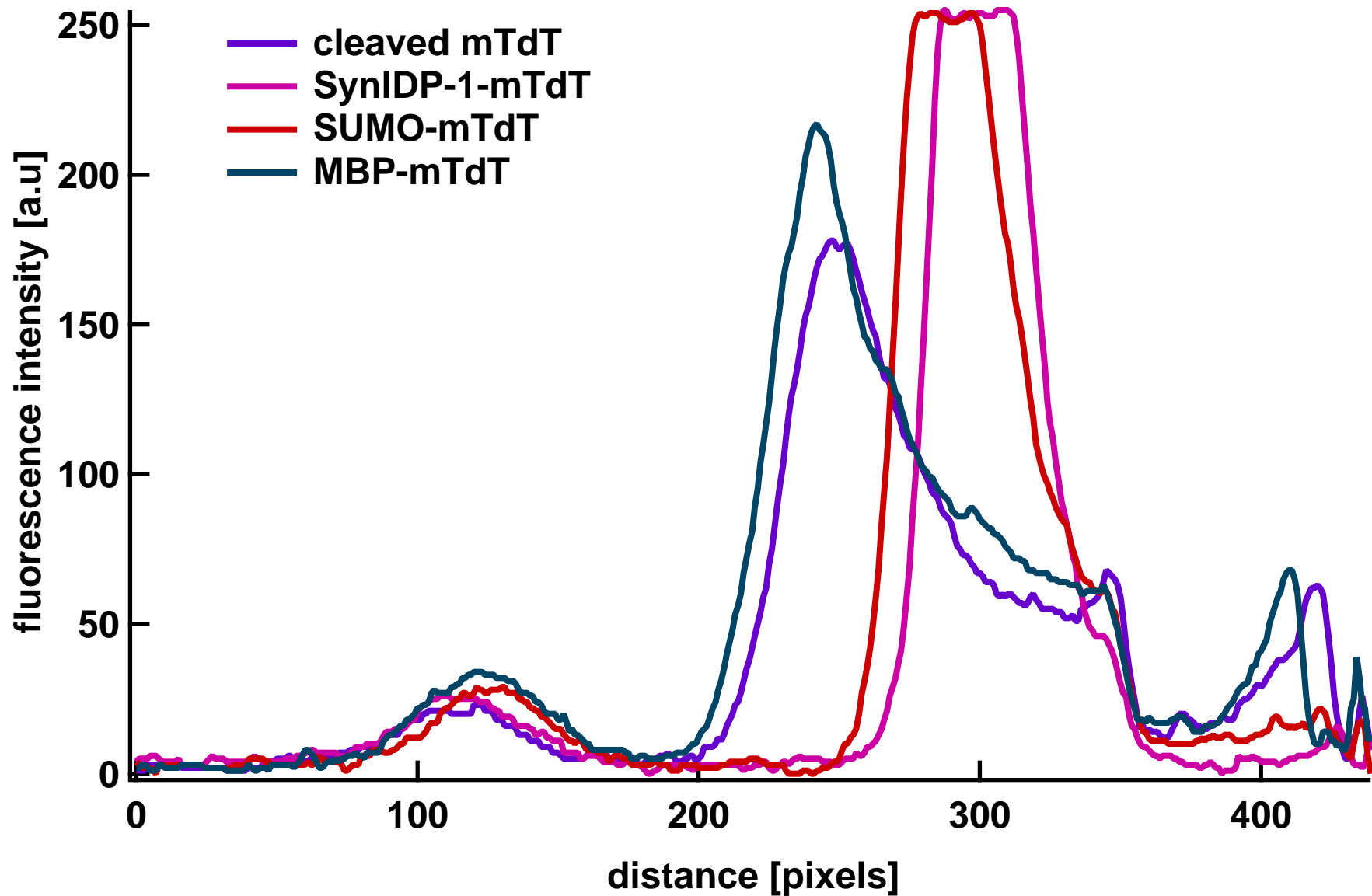

Supplement: Supplementary file 7 — Source Files [file 41467_2024_47519_MOESM7_ESM.zip › source files/TdT ASSAY-Figure 3/fluorescence.pdf]
